# Supplementary material for: Downregulation of semaphorin 4A in keratinocytes reflects the features of non-lesional psoriasis
Source: eLife. 2024 Dec 31;13:RP97654. doi: 10.7554/eLife.97654 (PMC11687936; doi:10.7554/eLife.97654)

Unedited blot images

Downregulation of Semaphorin 4A in keratinocytes reflects the features of non-lesional psoriasis

Figure 6, C and D

Figure 6

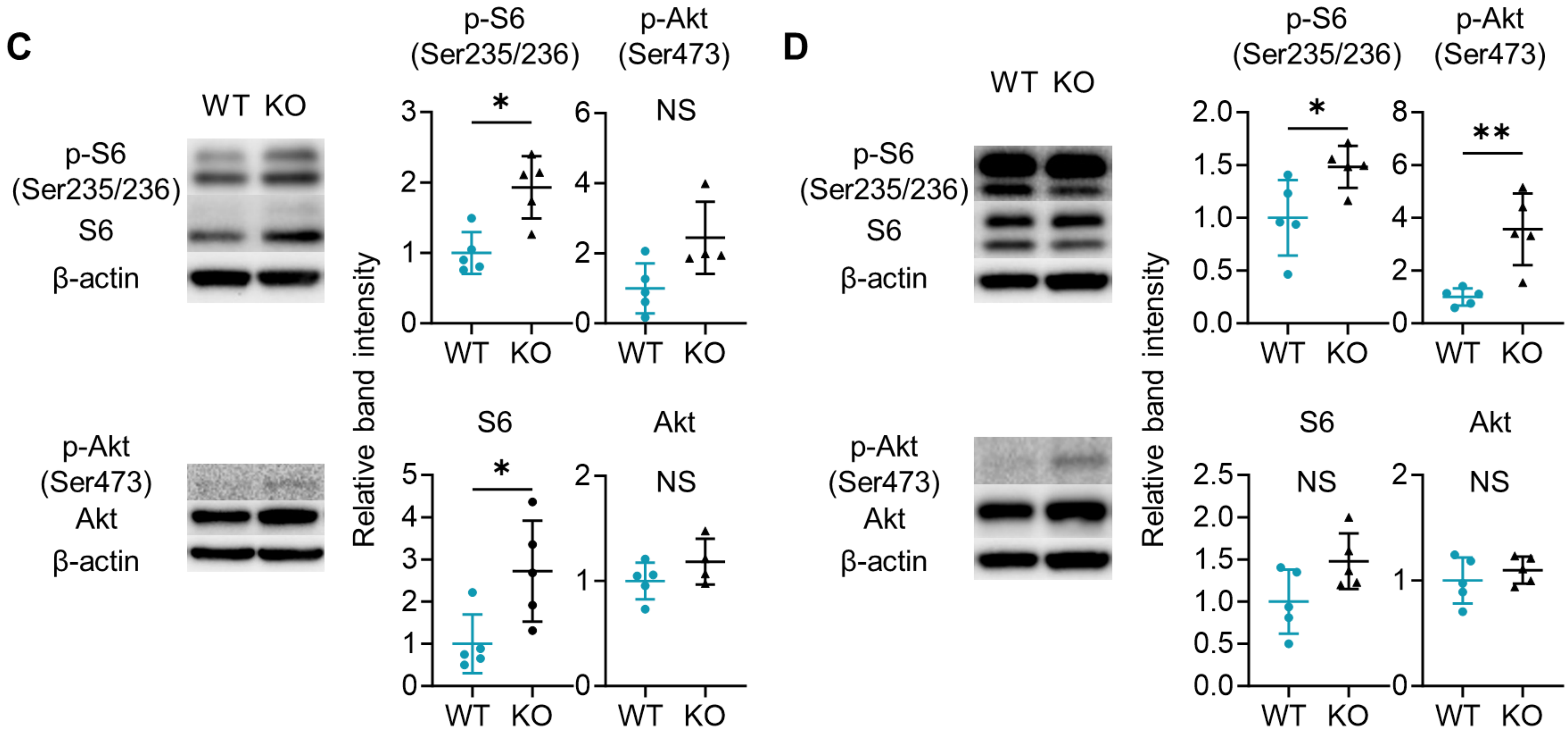
C
WT KO
p-S6
(Ser235/236)
S6
β-actin
p-Akt
(Ser473)
Akt
β-actin
Relative band intensity
p-S6
(Ser235/236)
p-Akt
(Ser473)
S6
Akt
*
NS
*
NS
WT
KO
D
WT KO
p-S6
(Ser235/236)
S6
β-actin
p-Akt
(Ser473)
Akt
β-actin
Relative band intensity
p-S6
(Ser235/236)
p-Akt
(Ser473)
S6
Akt
*
**
NS
NS
WT
KO

Full unedited blot for Figure 6C p-S6

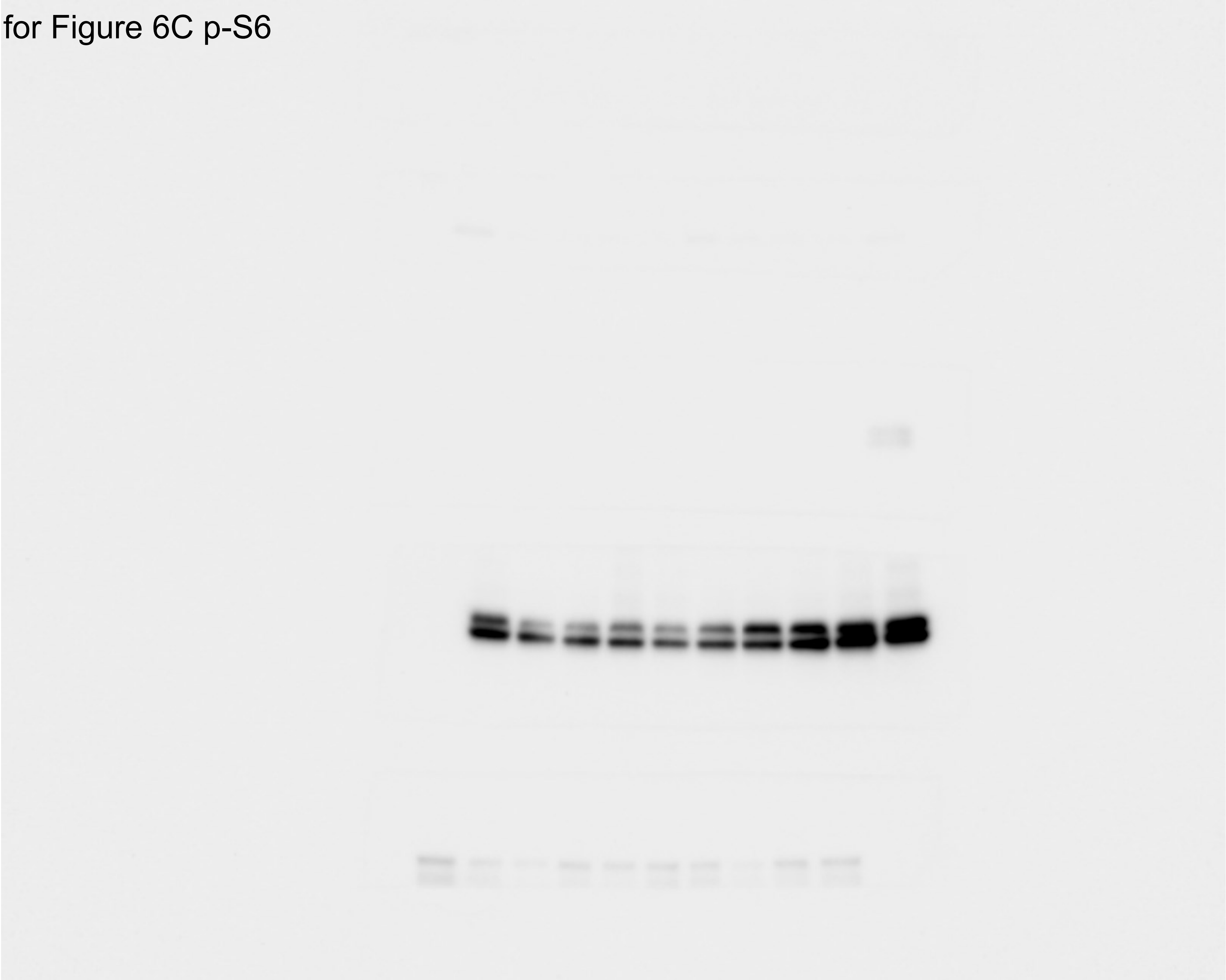

Full unedited blot for Figure 6C p-S6

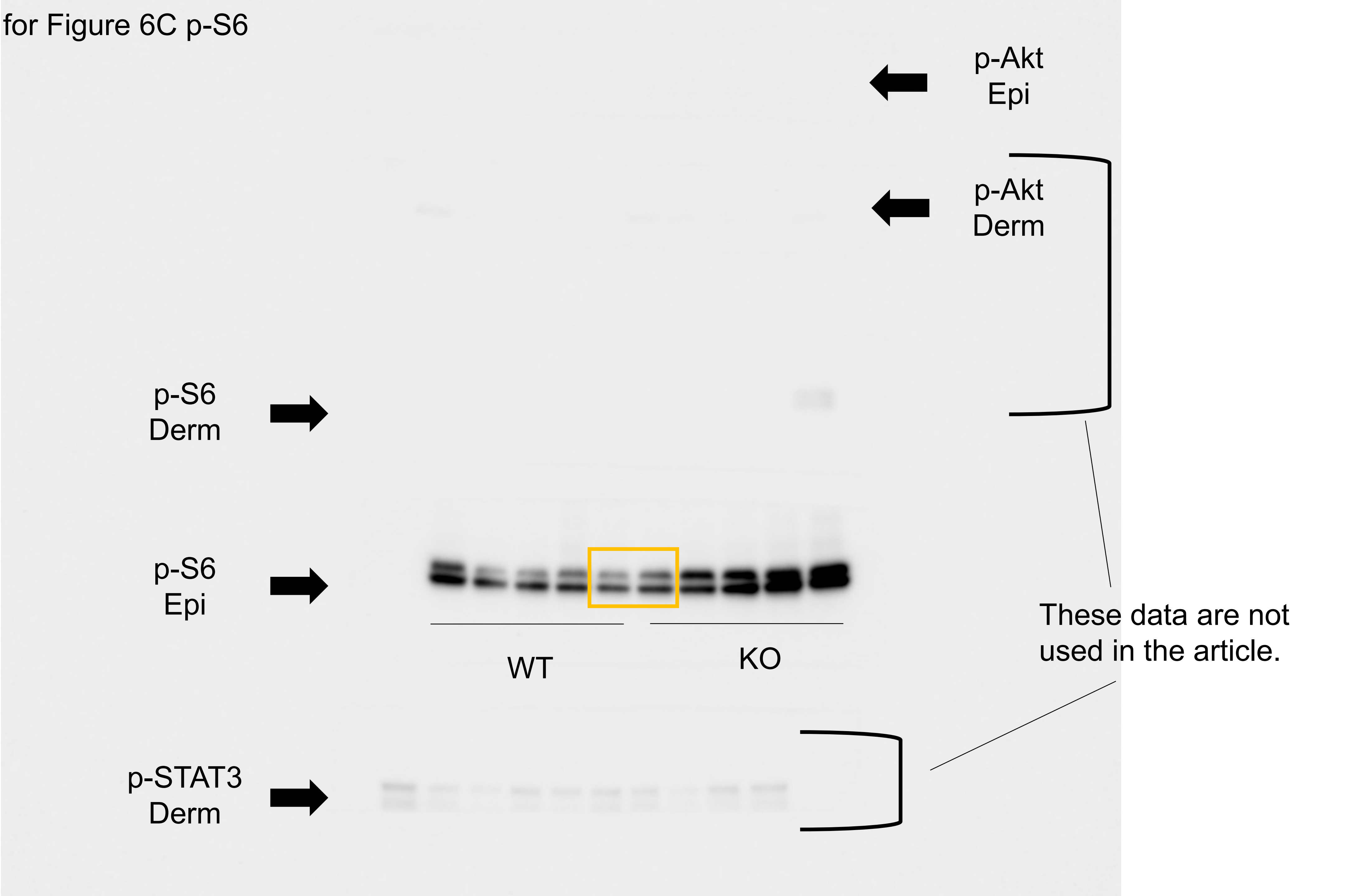

Full unedited blot for Figure 6C S6

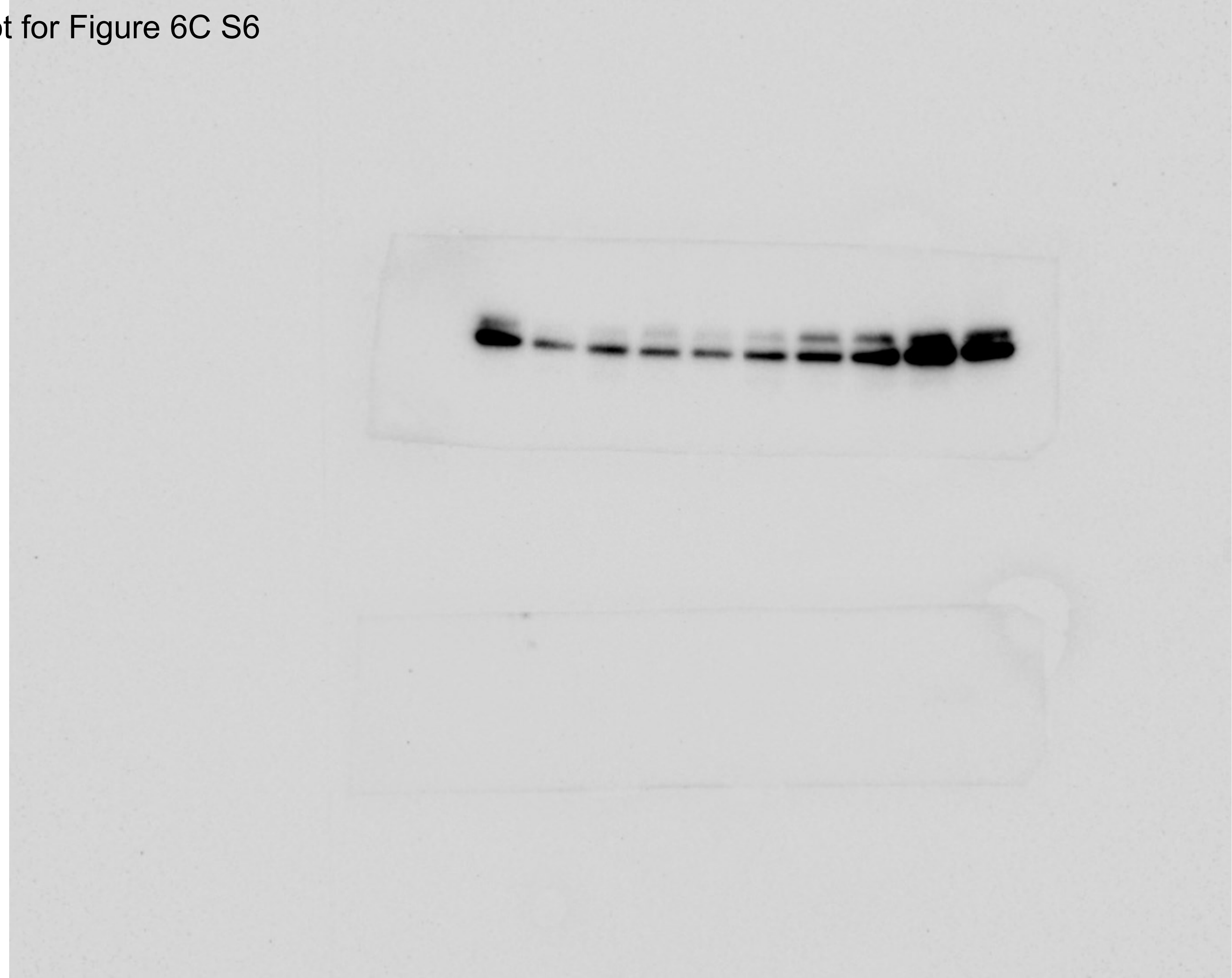

Full unedited blot for Figure 6C S6

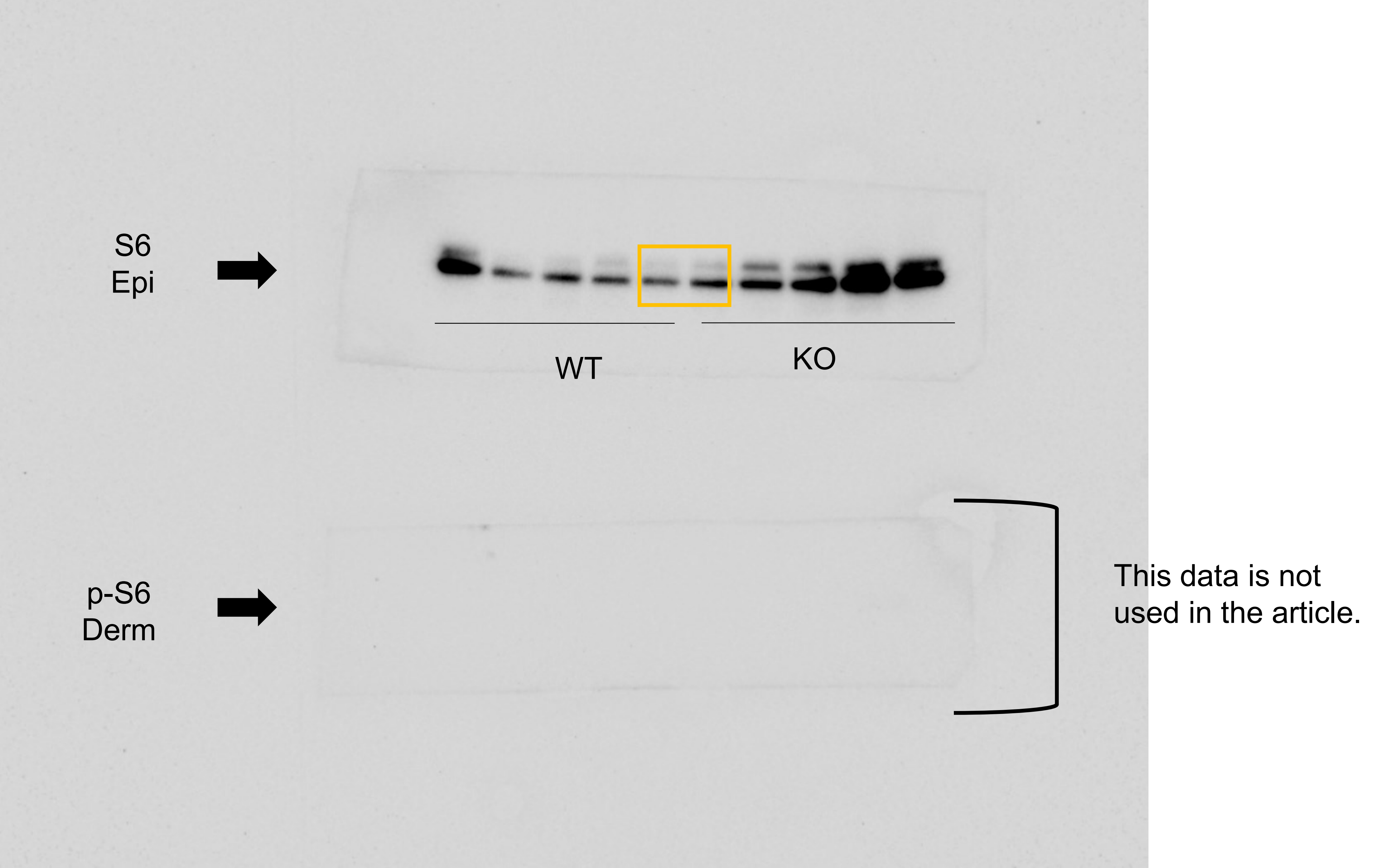

Full unedited blot for Figure 6C β-actin for p-S6, S6

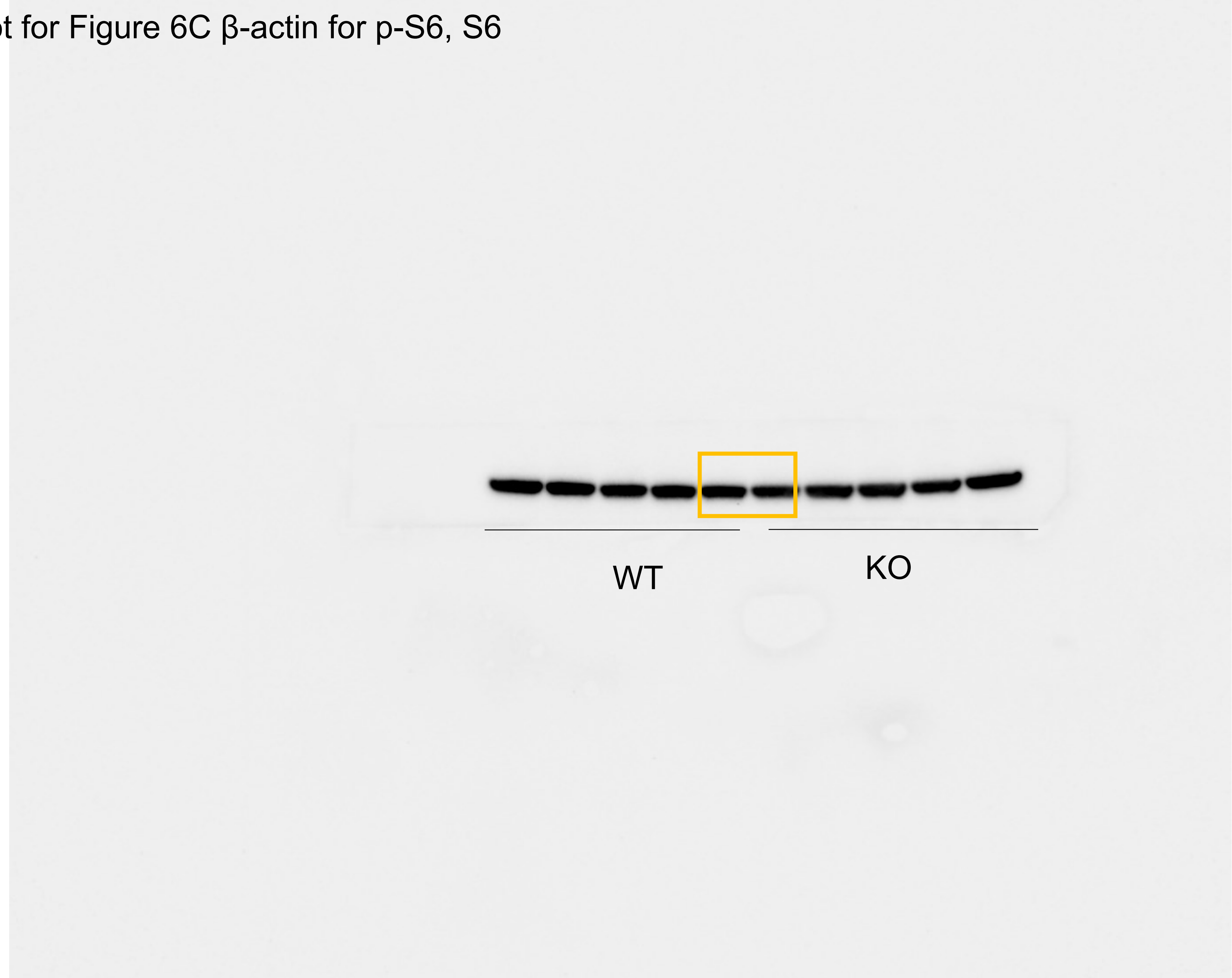

Full unedited blot for Figure 6C β-actin for p-S6, S6

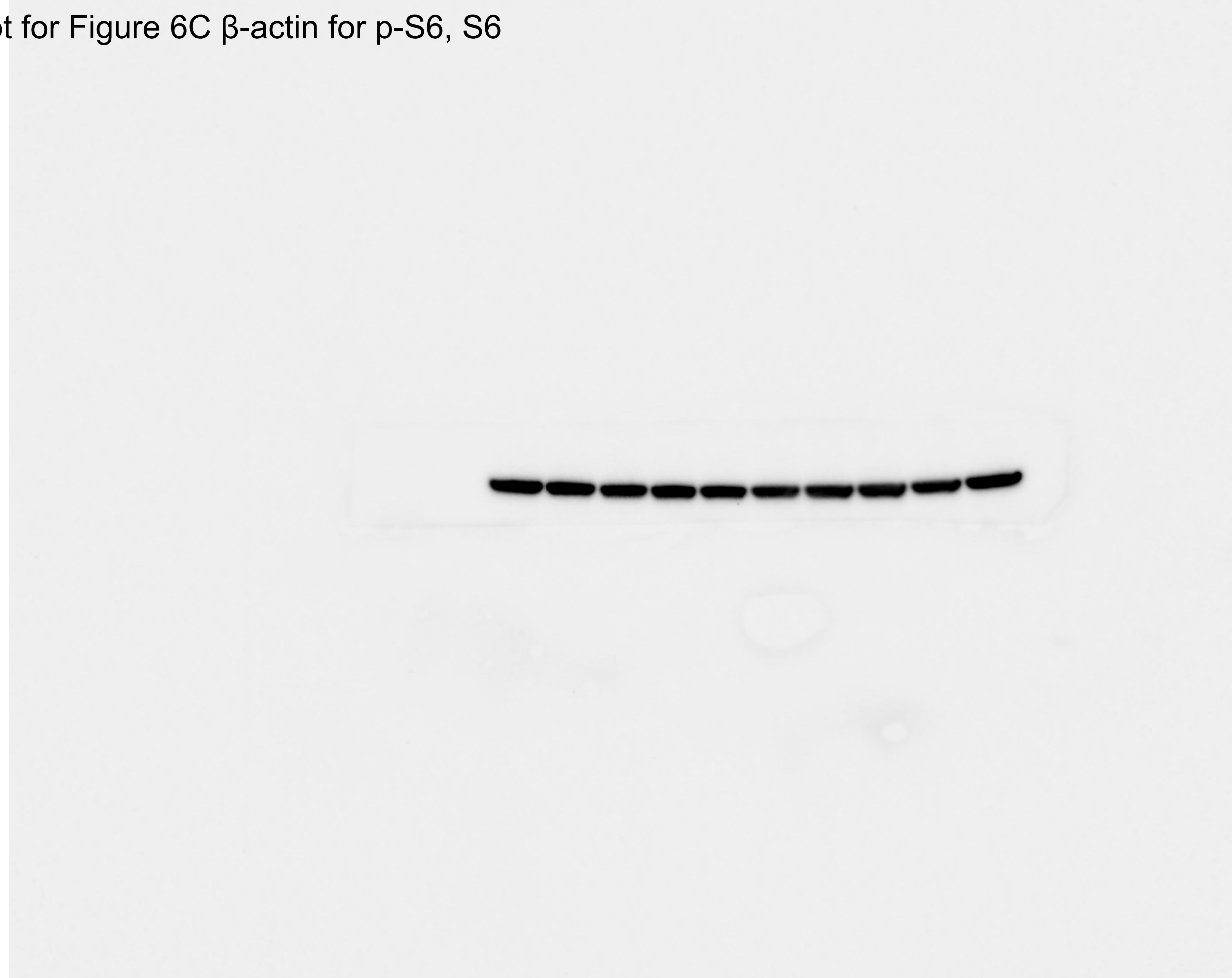

Full unedited blot for Figure 6C p-Akt

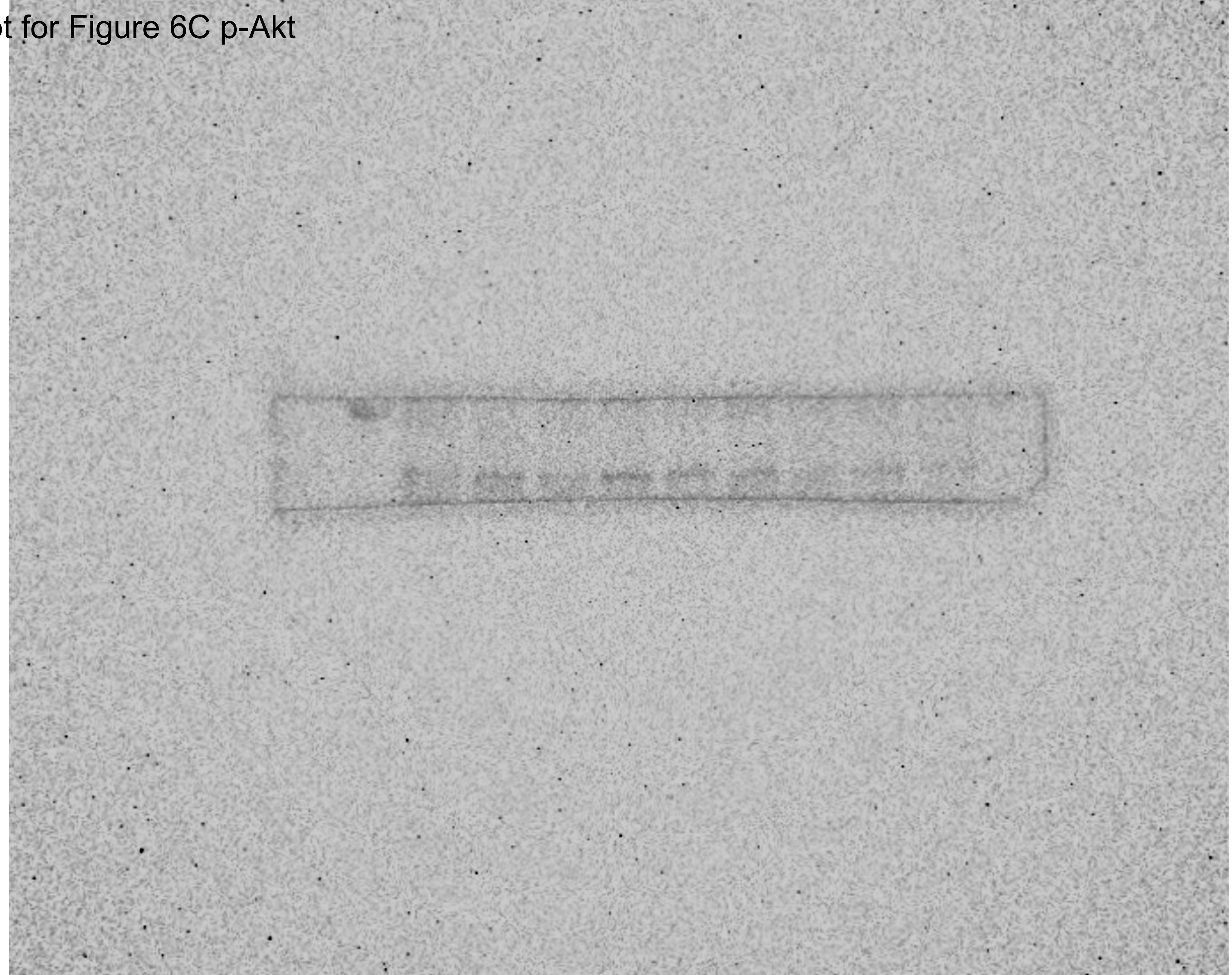

Full unedited blot for Figure 6C p-Akt

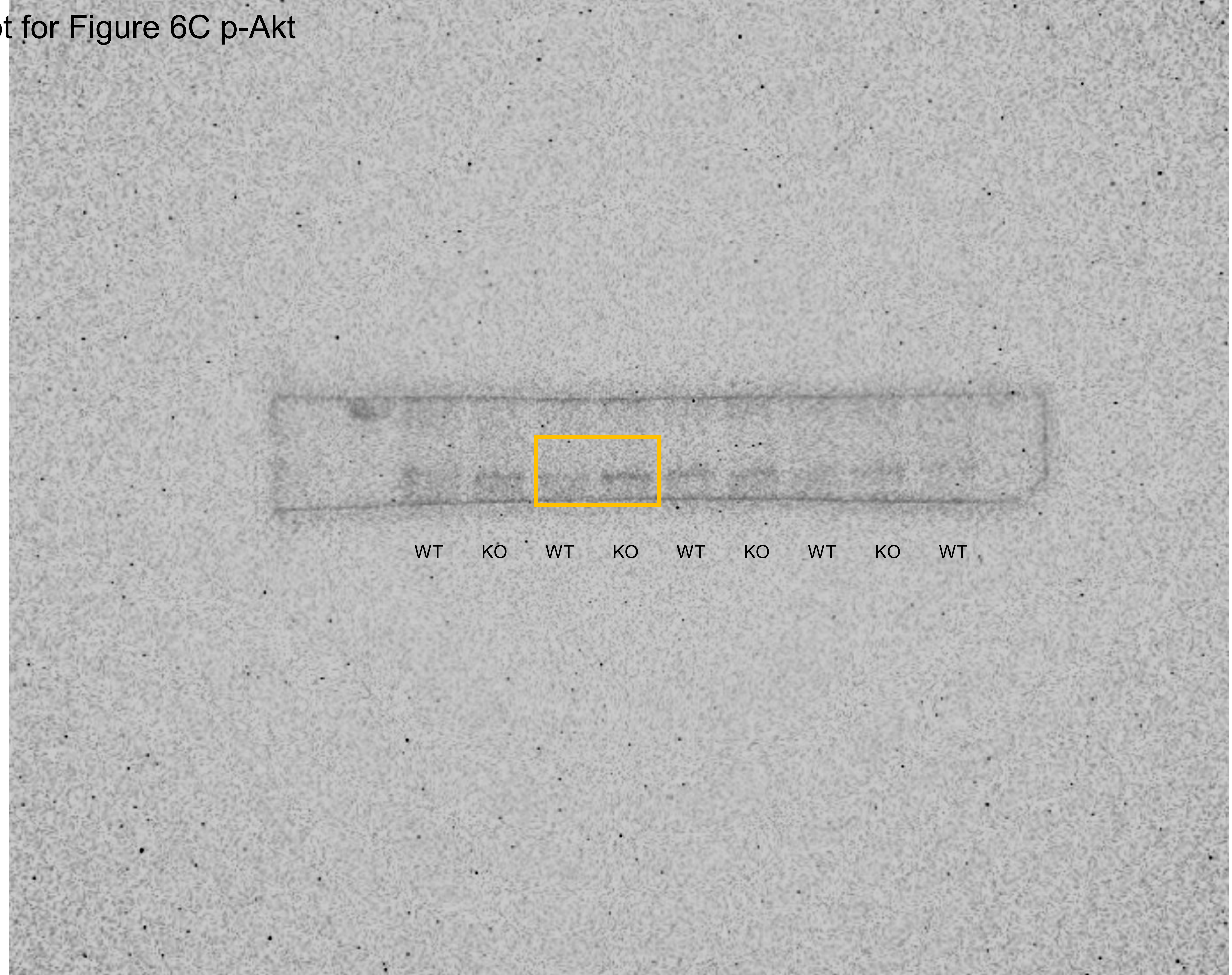

Full unedited blot for Figure 6C Akt

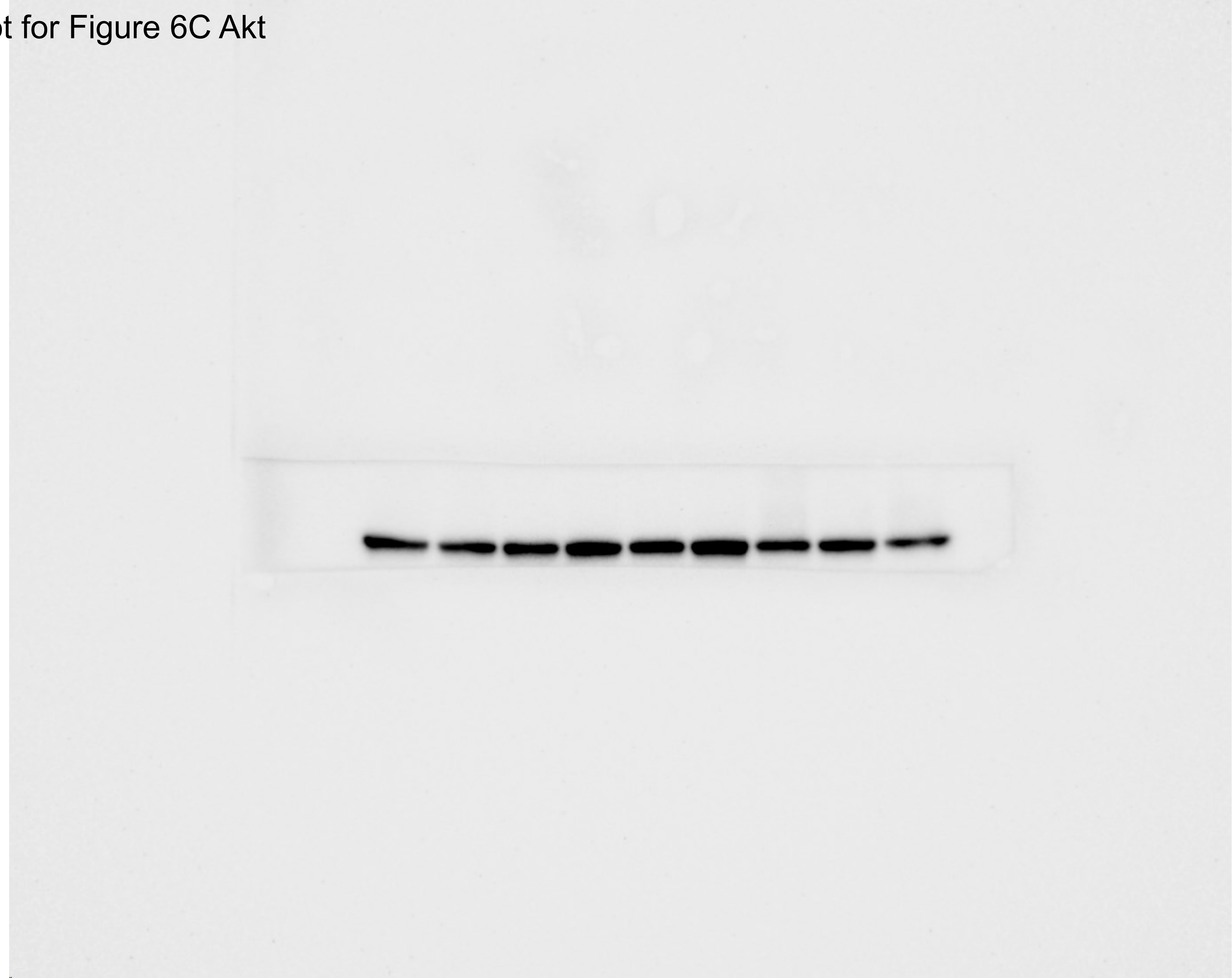

Full unedited blot for Figure 6C Akt

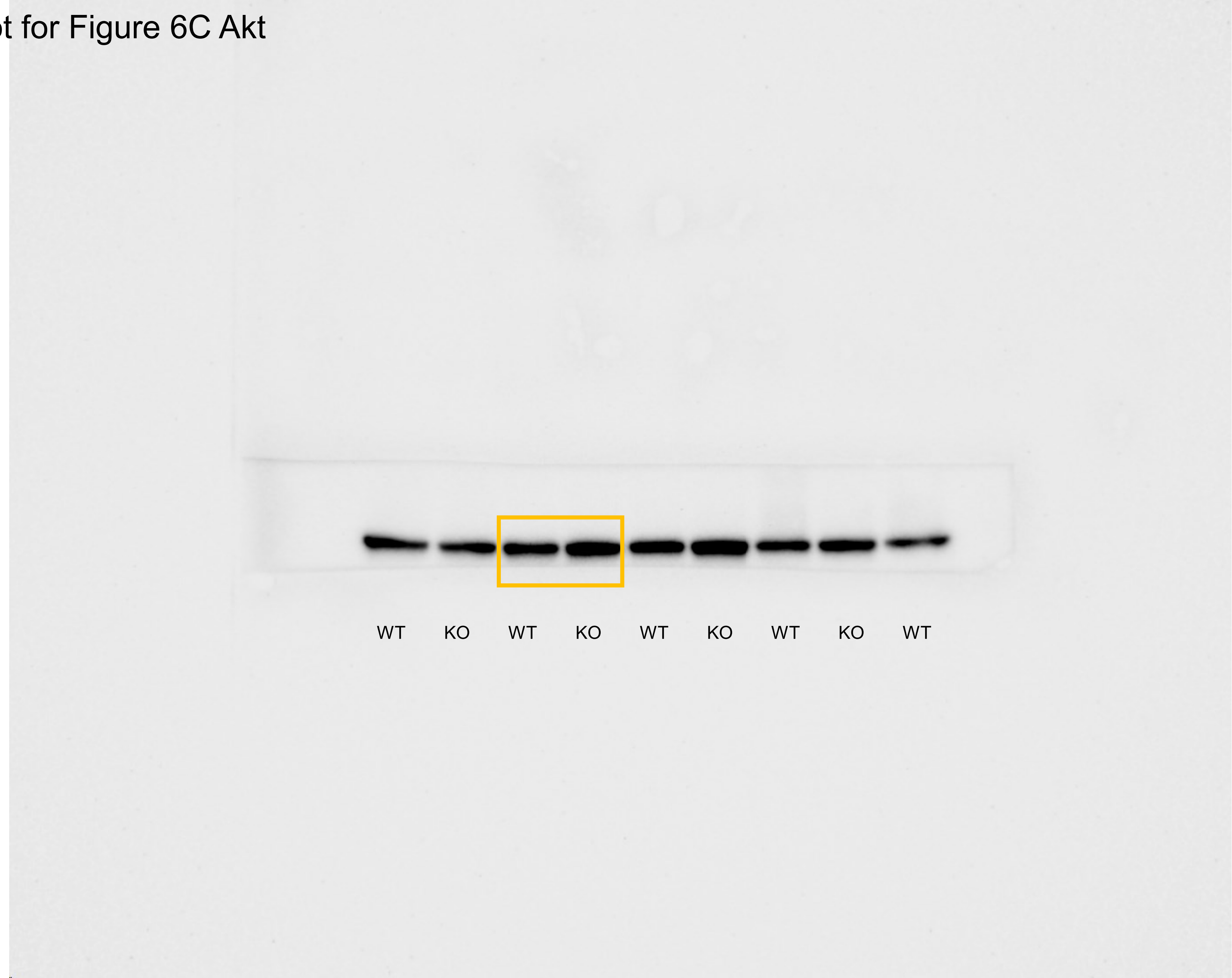

Full unedited blot for Figure 6C β-actin for p-Akt and Akt

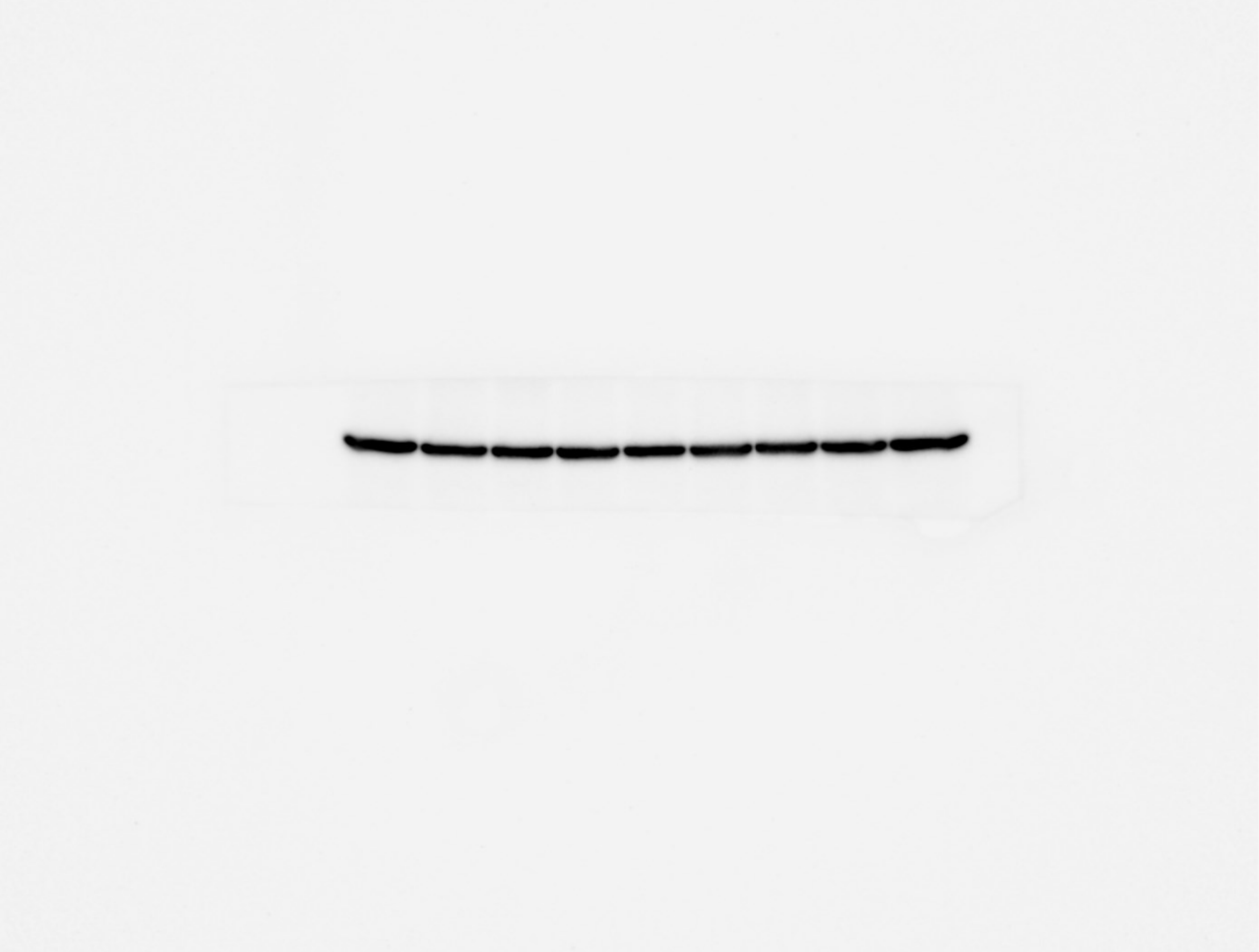

Full unedited blot for Figure 7C β-actin for p-Akt and Akt

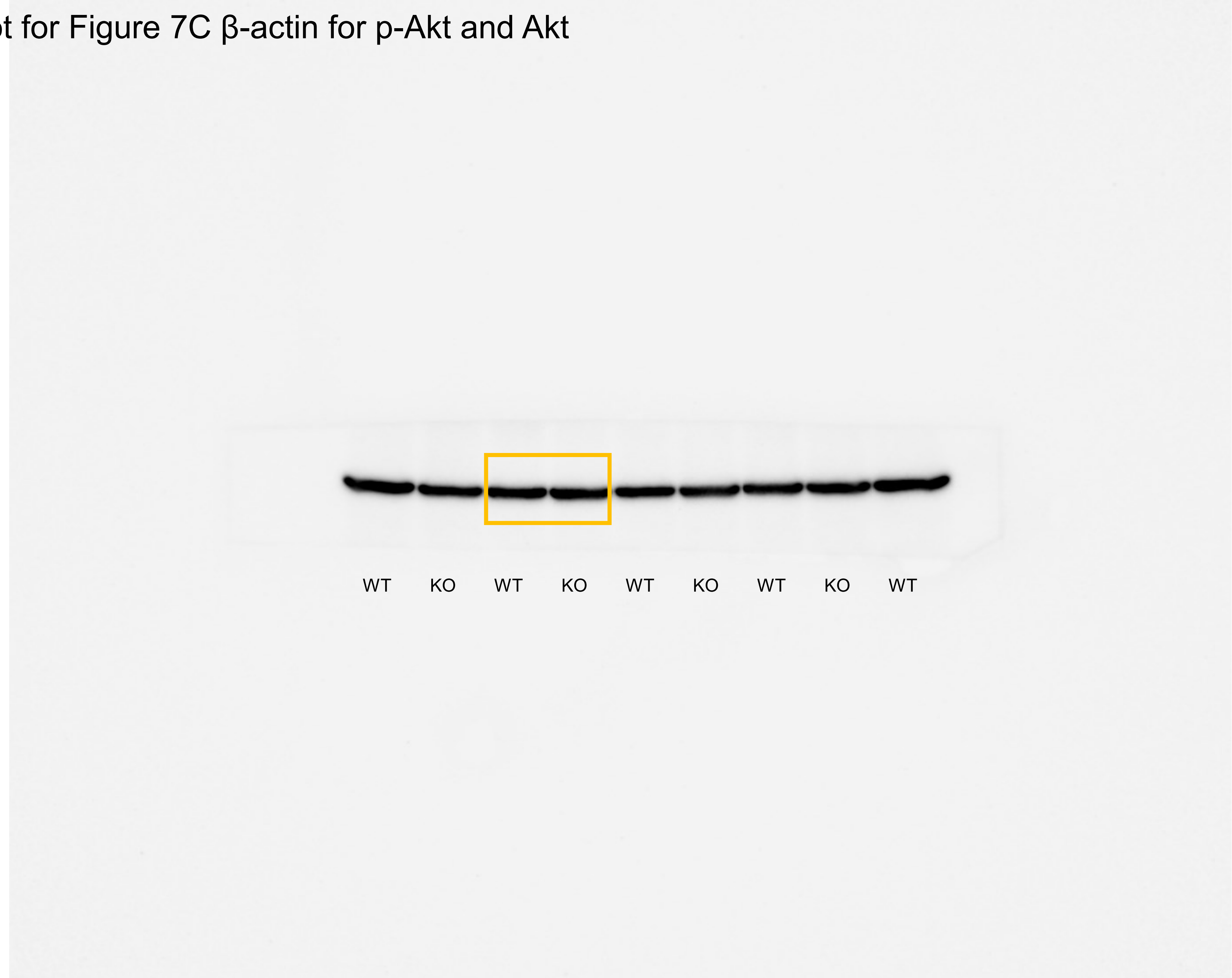

Full unedited blot for Figure 6D p-Akt and p-S6

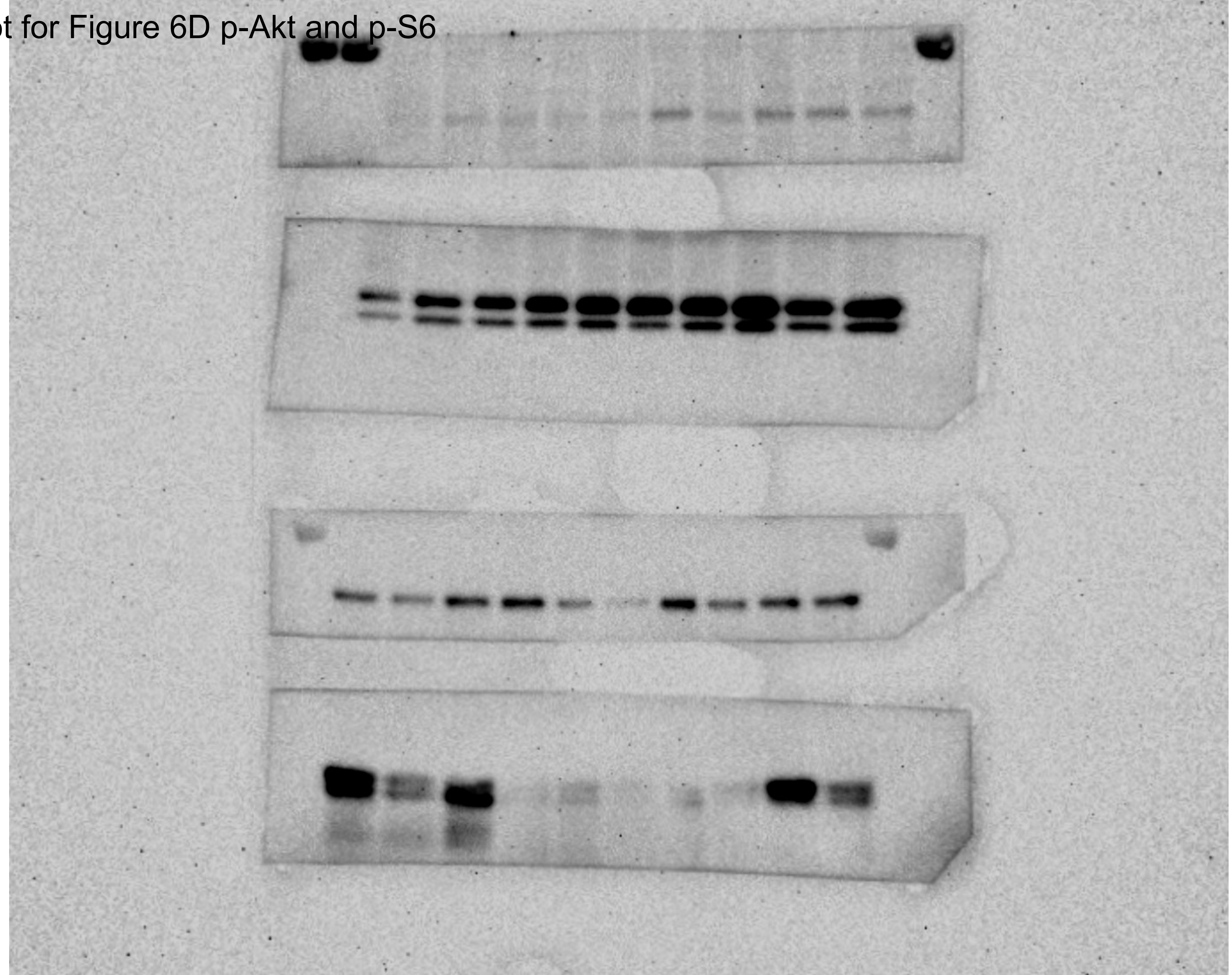

Full unedited blot for Figure 6D p-Akt and p-S6

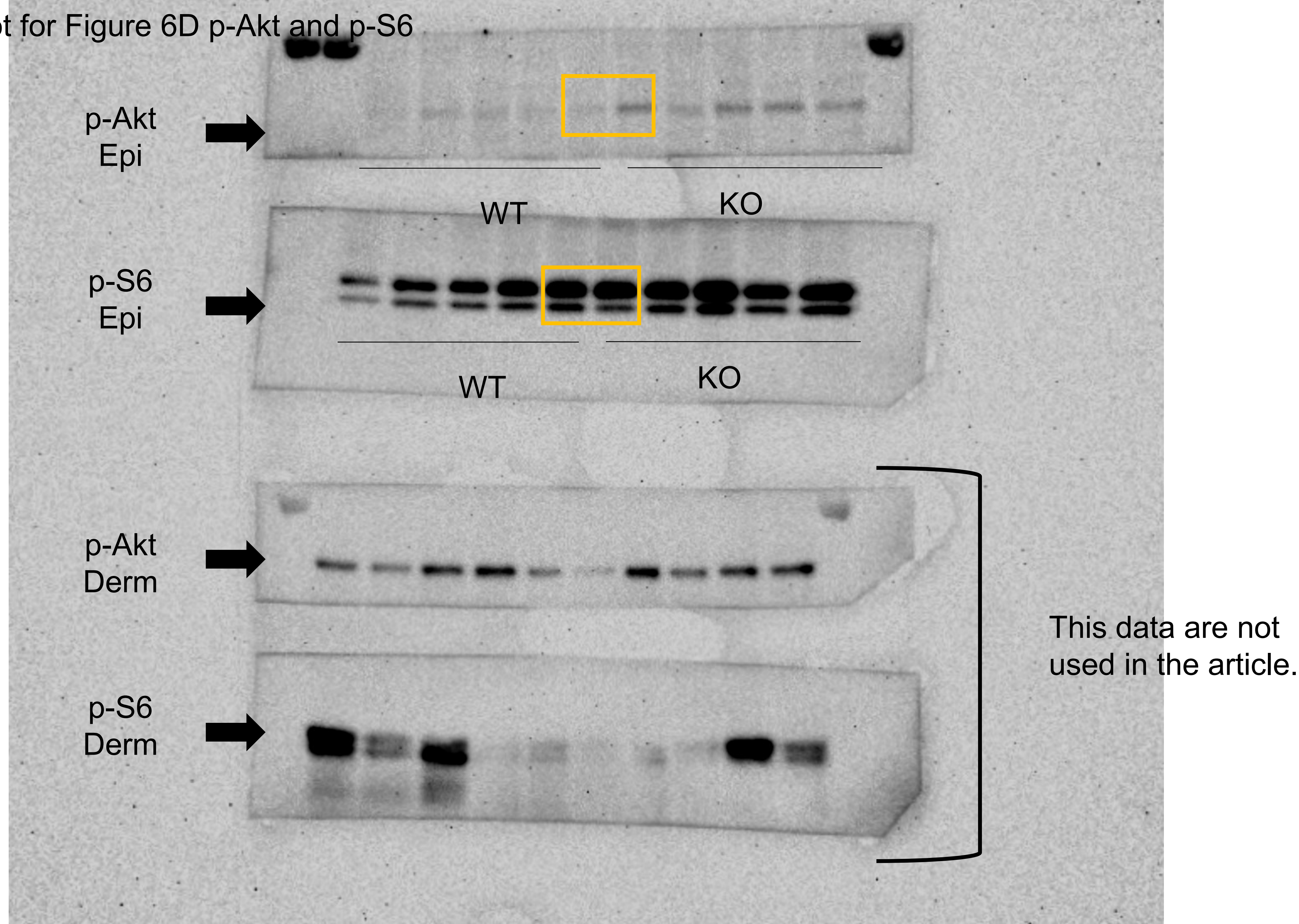

Full unedited blot for Figure 6D S6

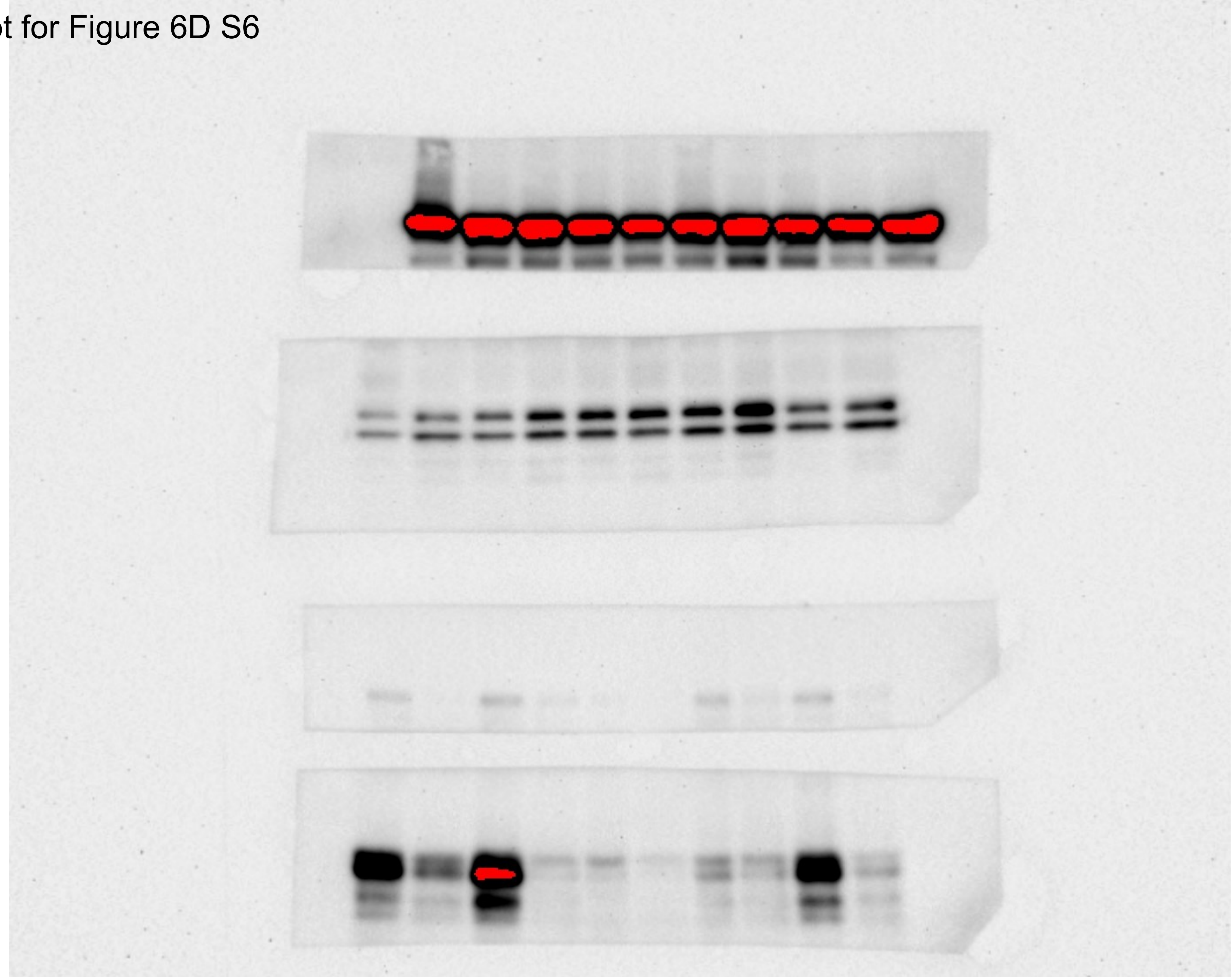

Full unedited blot for Figure 6D S6

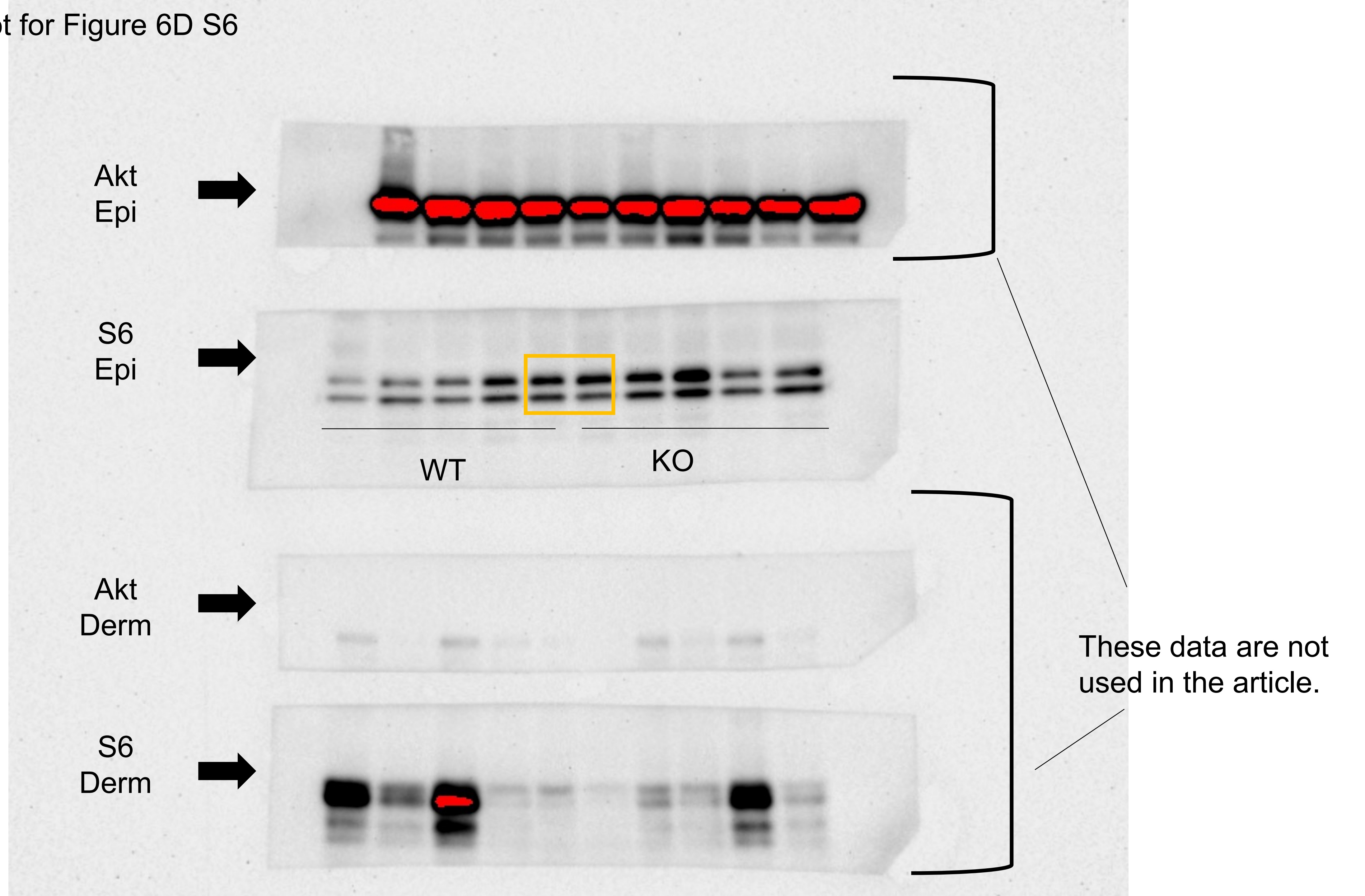

Full unedited blot for Figure 6D Akt

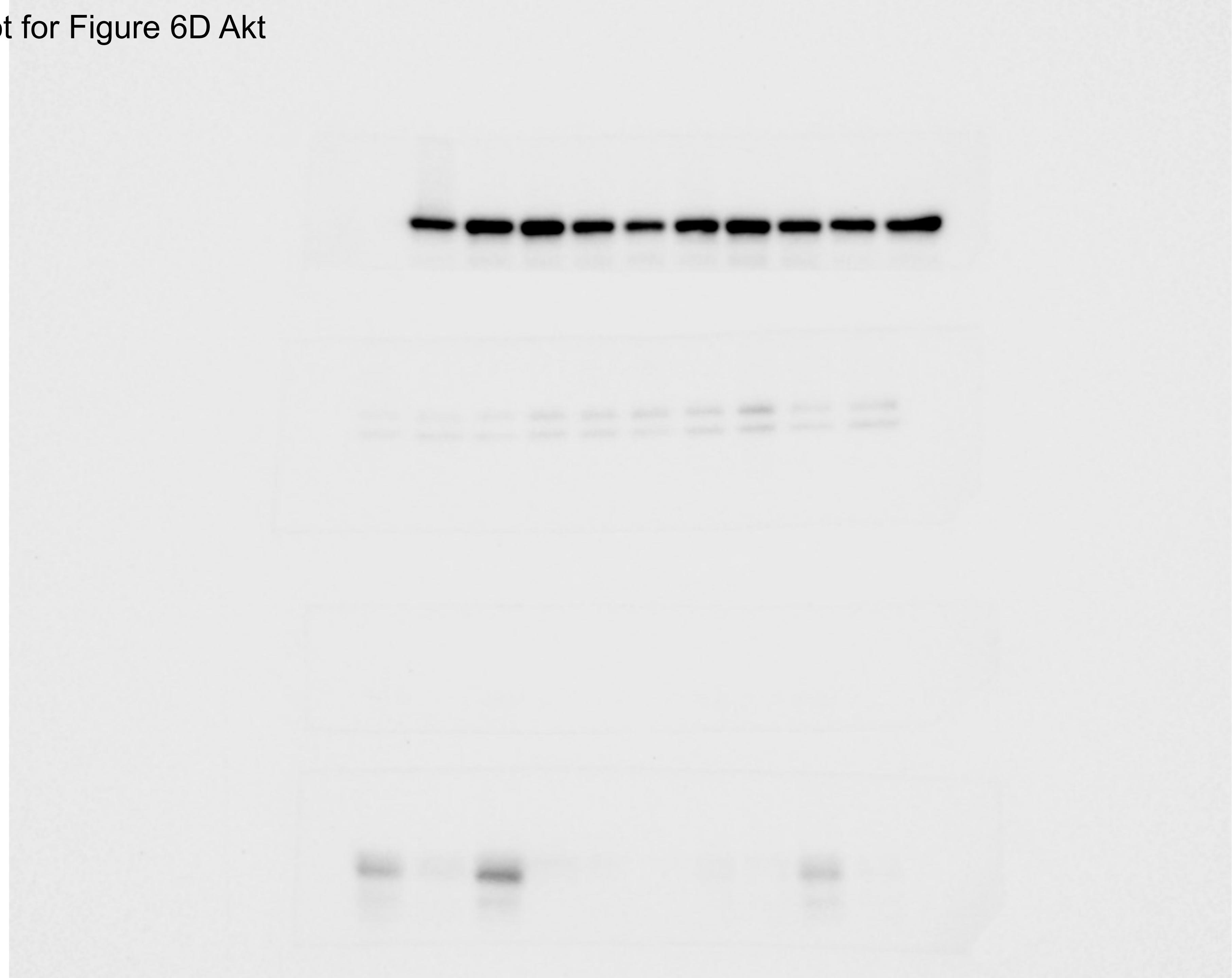

Full unedited blot for Figure 6D Akt

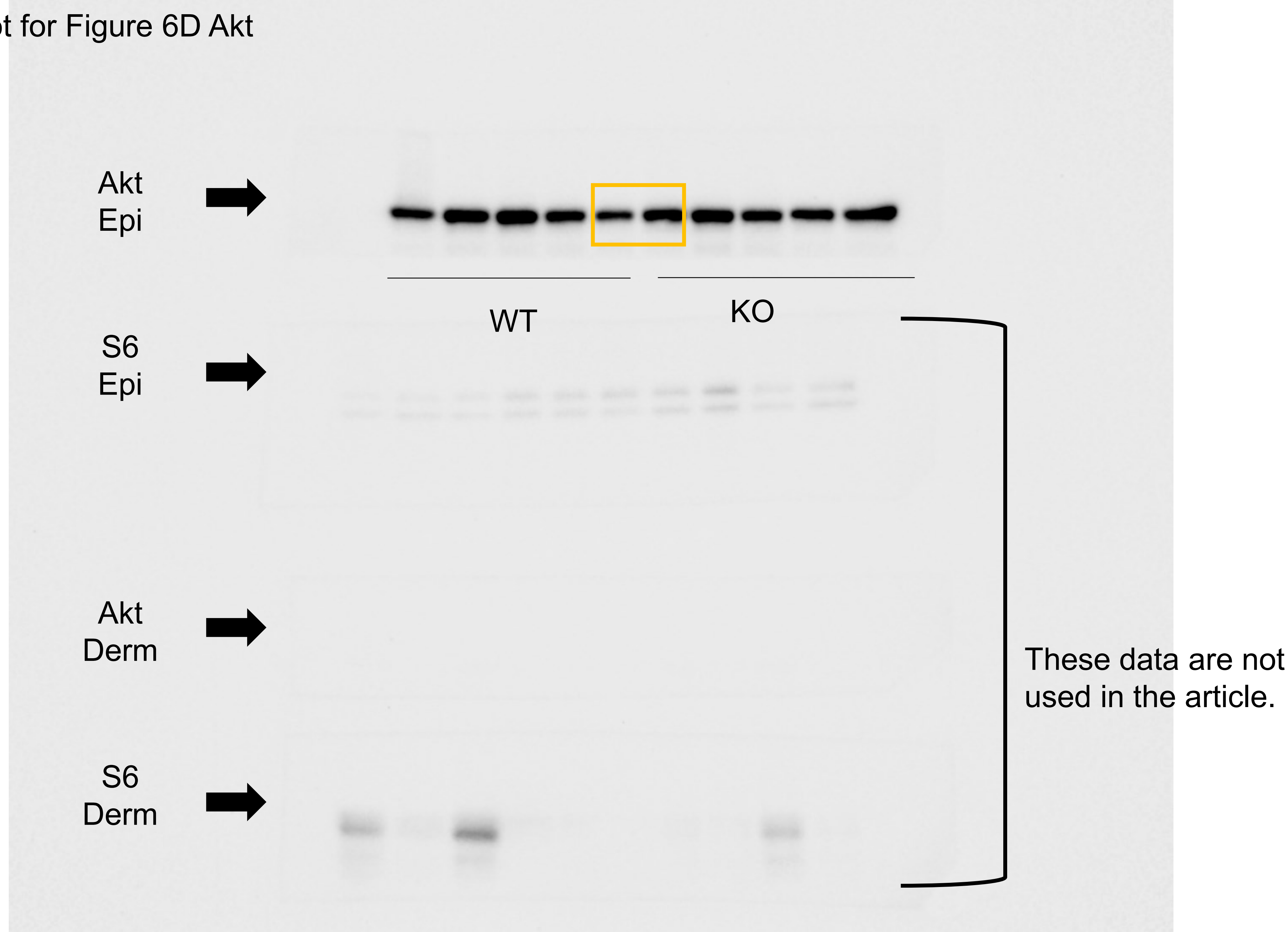

Full unedited blot for Figure 6D β-actin for p-S6, S6, p-Akt, and Akt

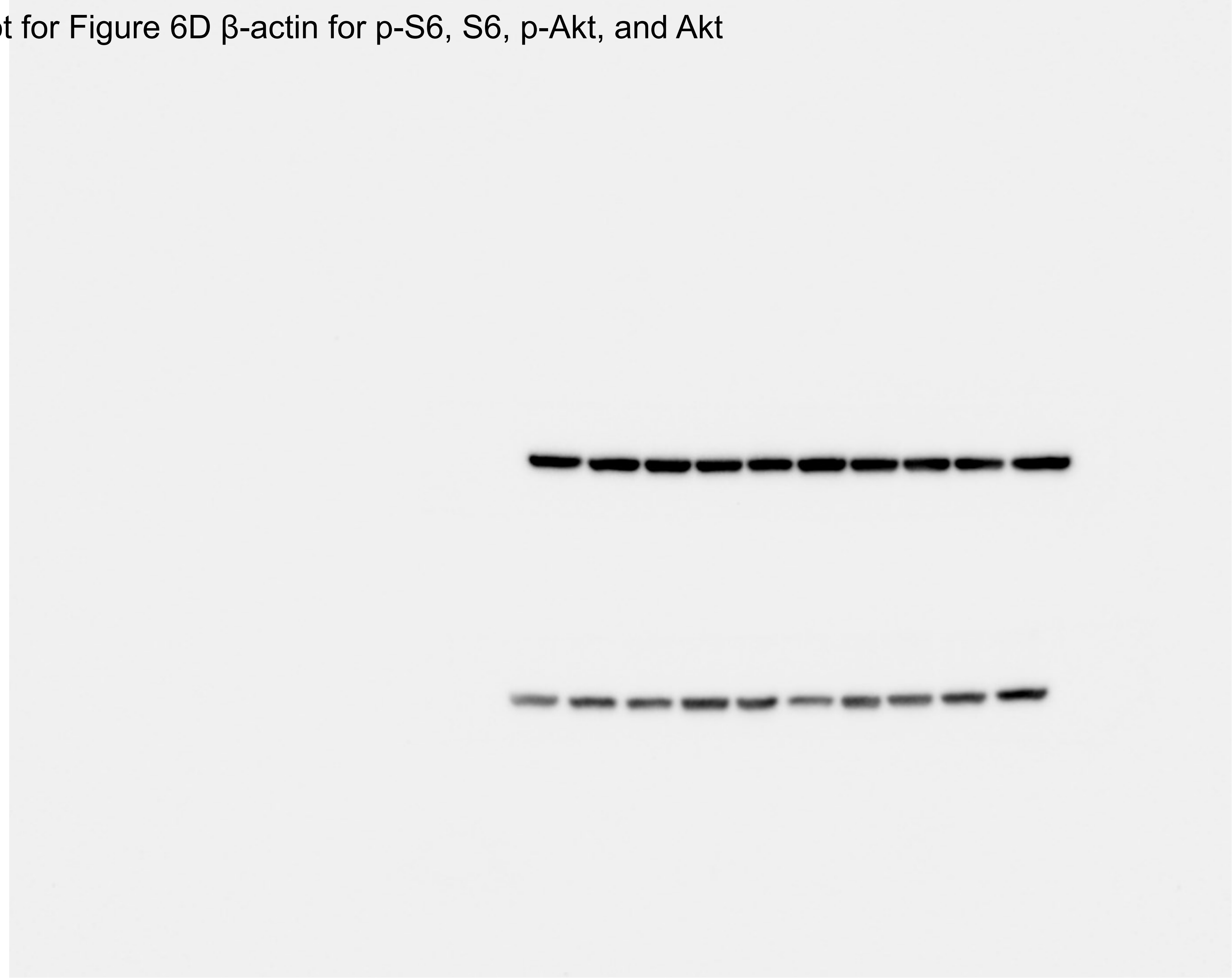

Full unedited blot for Figure 6D β-actin for p-S6, S6, p-Akt, and Akt

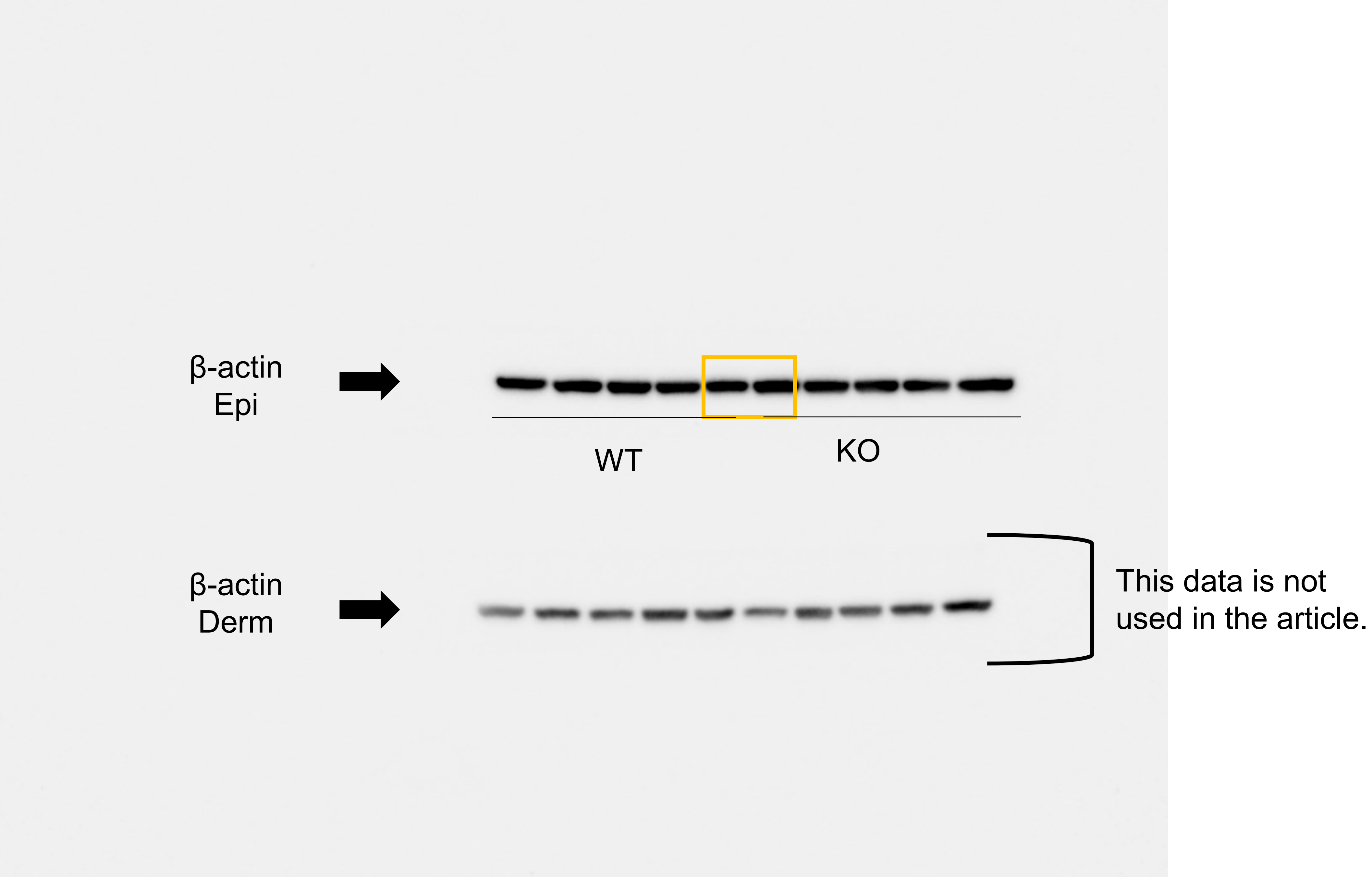

Supplement: Figure 6—source data 2. [file elife-97654-fig6-data2.zip › Figure 6-sourse data2.pdf]
