# Supplementary figures and images for: Downregulation of semaphorin 4A in keratinocytes reflects the features of non-lesional psoriasis
Source: eLife. 2024 Dec 31;13:RP97654. doi: 10.7554/eLife.97654 (PMC11687936; doi:10.7554/eLife.97654)

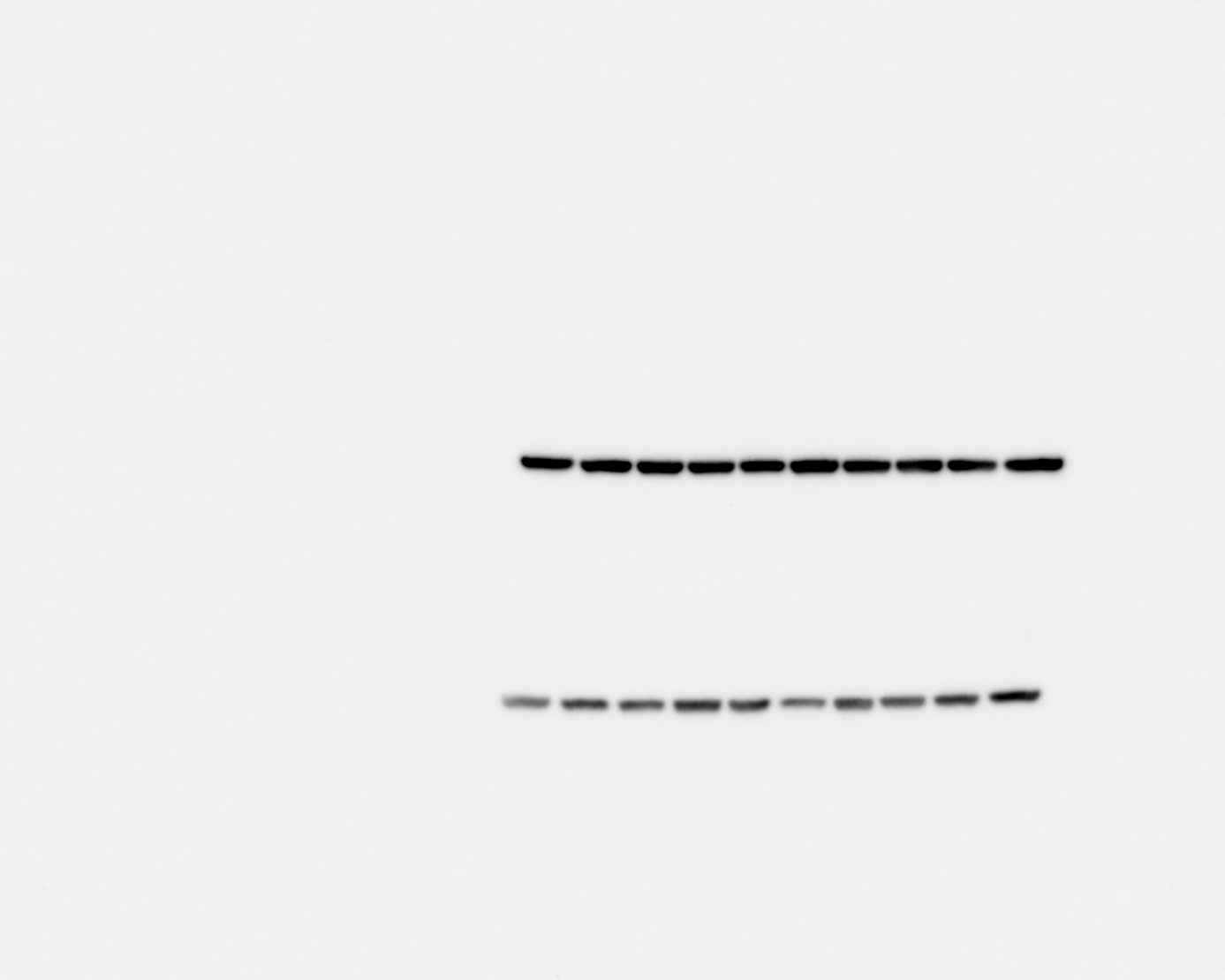

Supplement: Figure 6—source data 3. [file elife-97654-fig6-data3.zip › Figure 6-sourse data3/2022-09-01 14h47m56sFig6D_bactin.jpg]

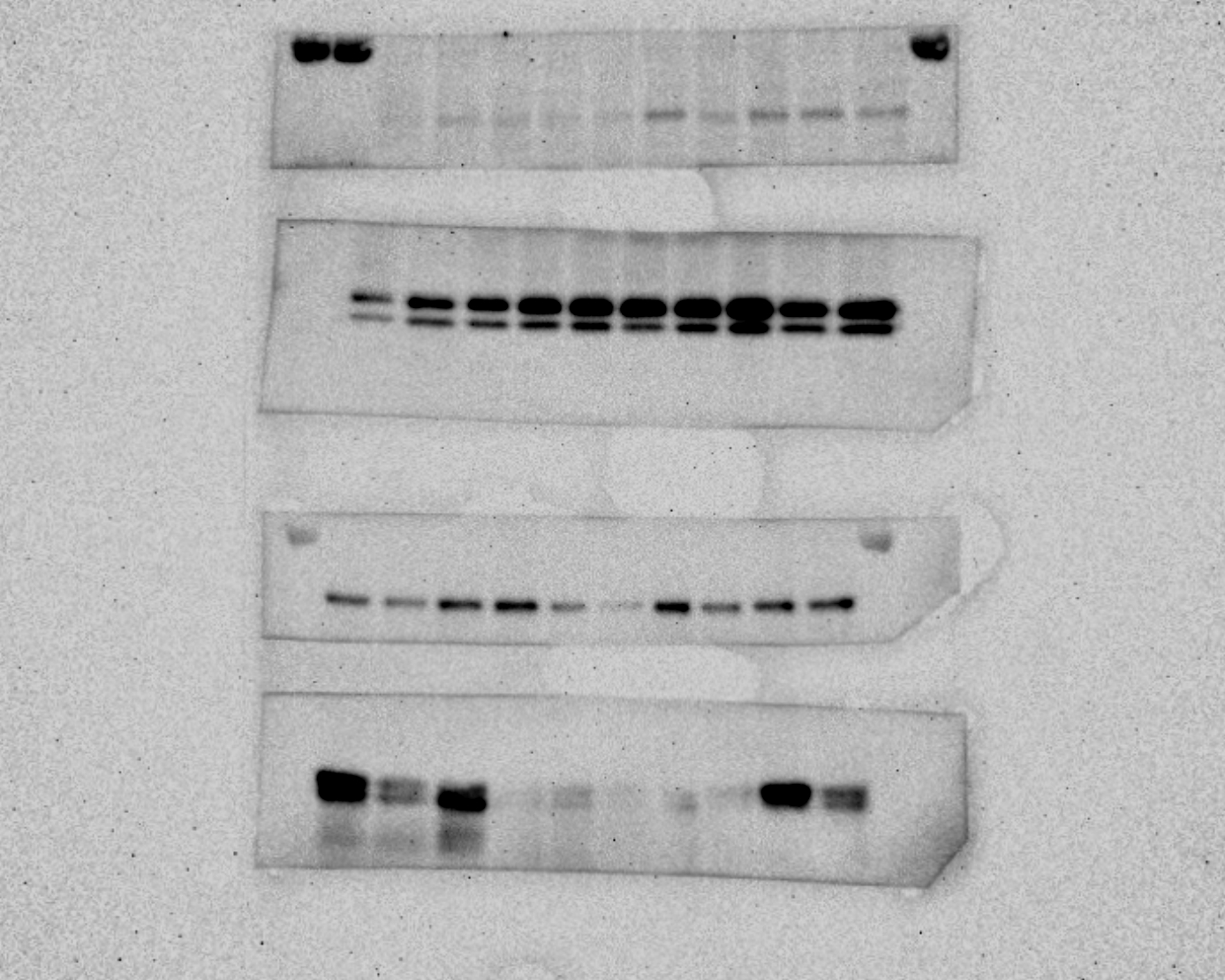

Supplement: Figure 6—source data 3. [file elife-97654-fig6-data3.zip › Figure 6-sourse data3/2022-09-01 Fig6D_p-Akt and p-S6.jpg]

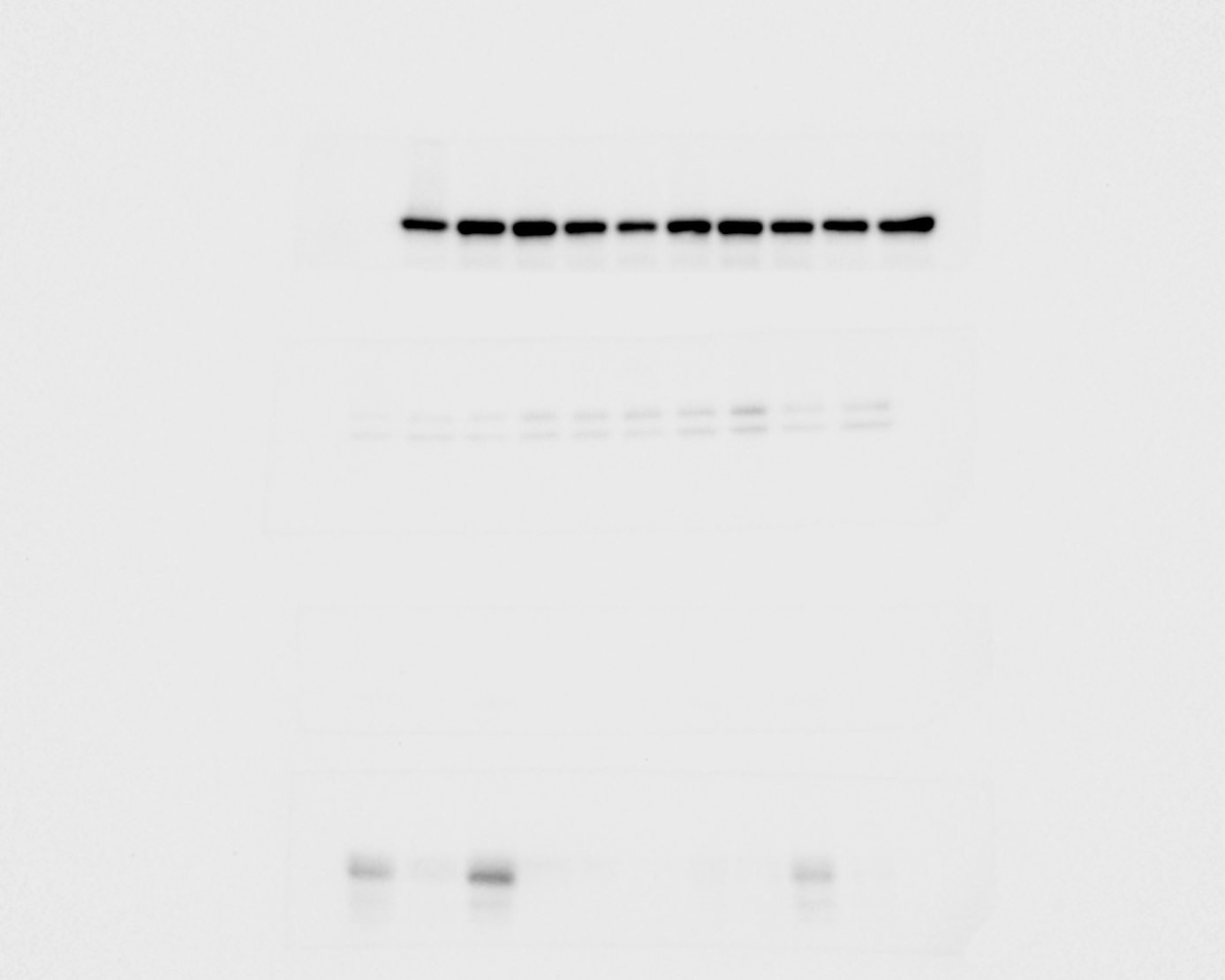

Supplement: Figure 6—source data 3. [file elife-97654-fig6-data3.zip › Figure 6-sourse data3/2022-09-02 Fig6D_Akt.jpg]

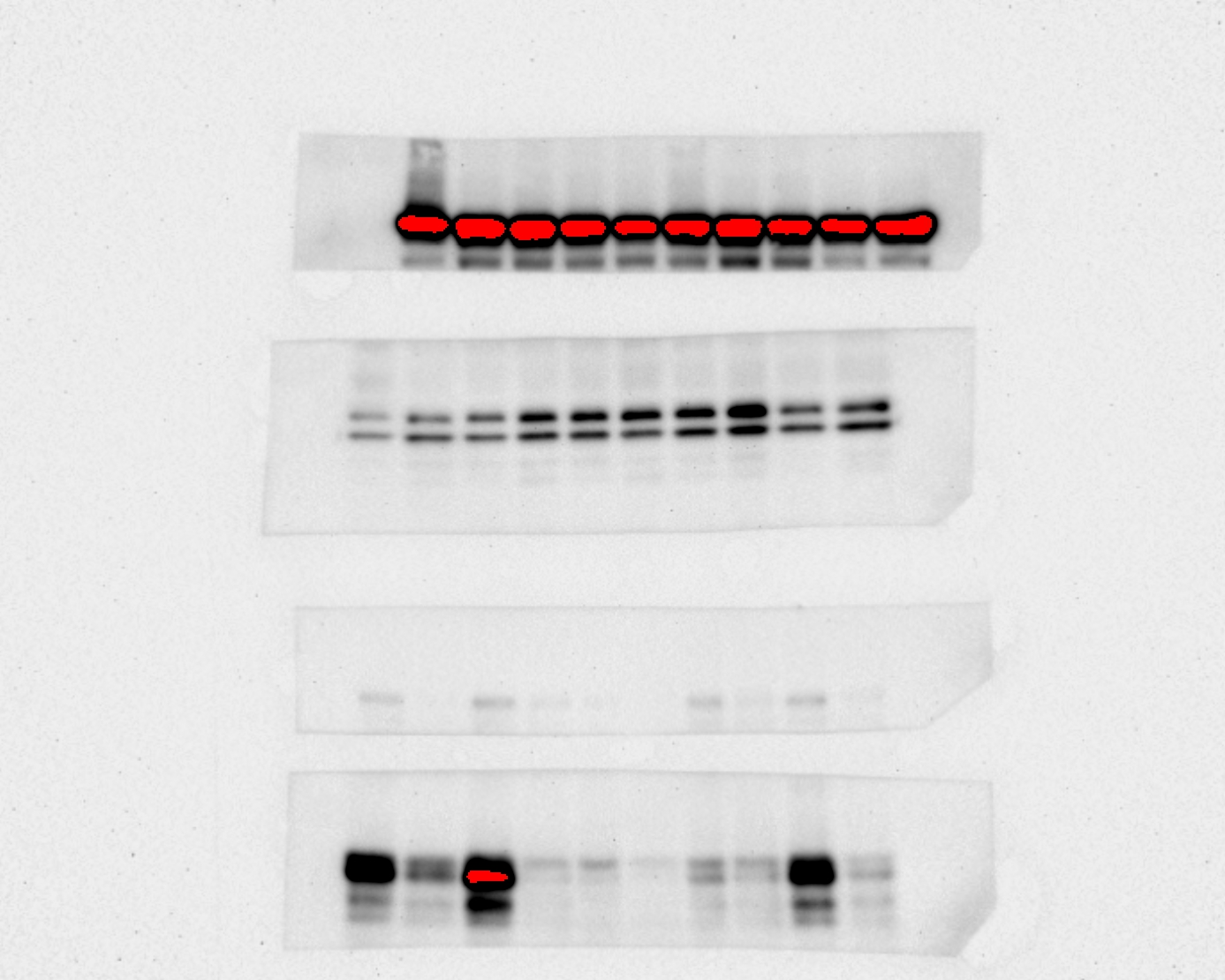

Supplement: Figure 6—source data 3. [file elife-97654-fig6-data3.zip › Figure 6-sourse data3/2022-09-02 Fig6D_S6.jpg]

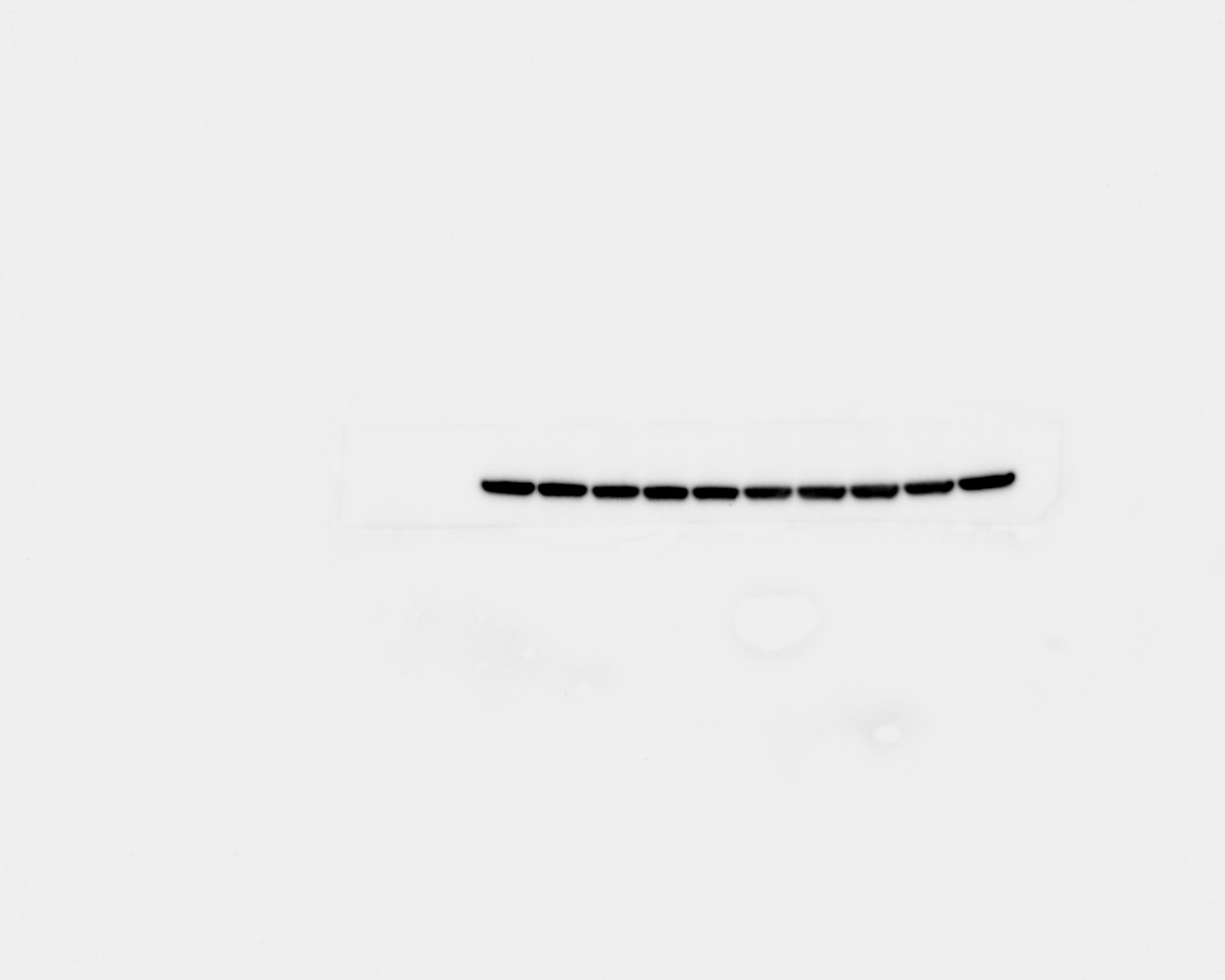

Supplement: Figure 6—source data 3. [file elife-97654-fig6-data3.zip › Figure 6-sourse data3/2022-09-14 Fig6C_bactin for p-S6 and S6.jpg]

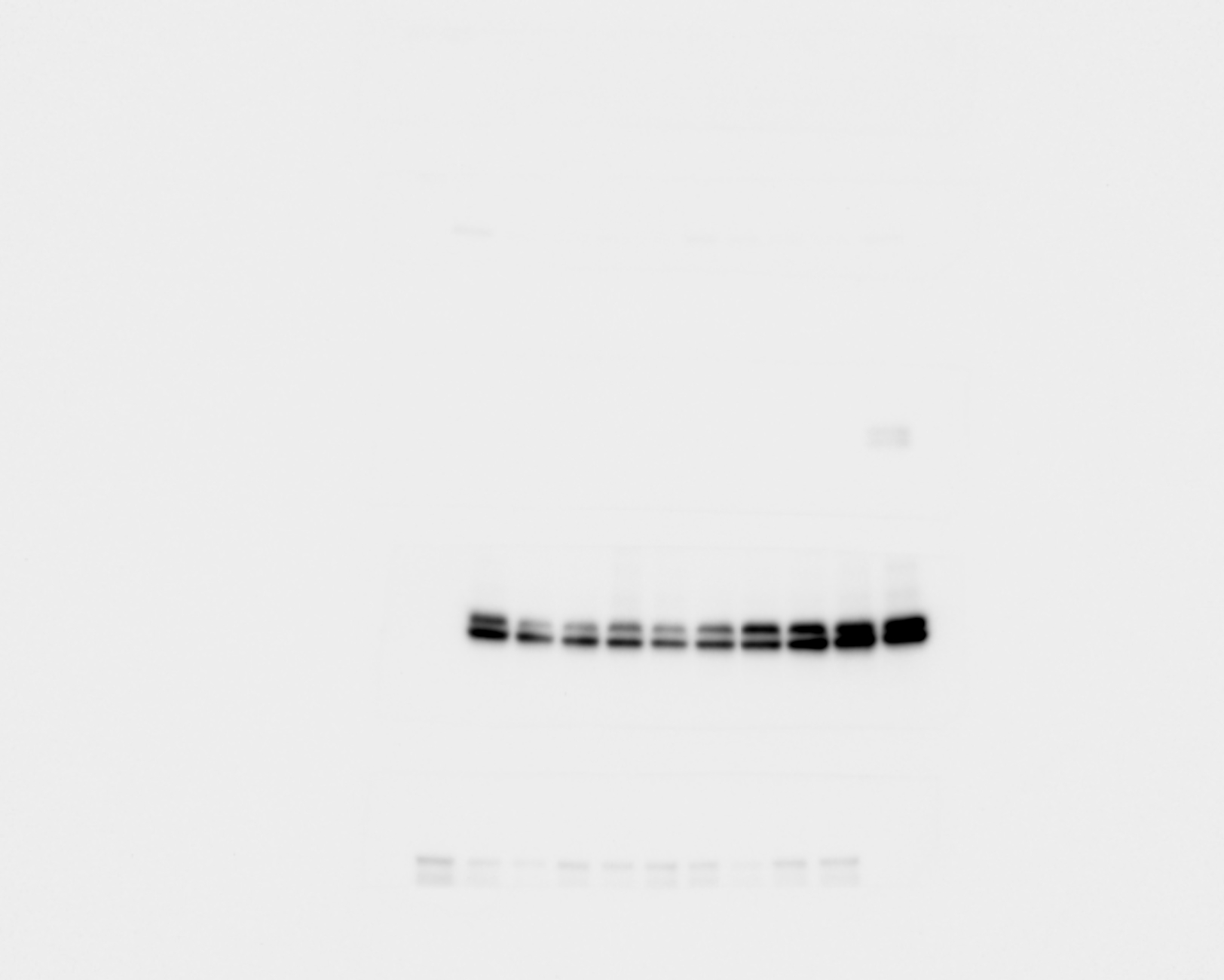

Supplement: Figure 6—source data 3. [file elife-97654-fig6-data3.zip › Figure 6-sourse data3/2022-09-14 Fig6C_p-S6.jpg]

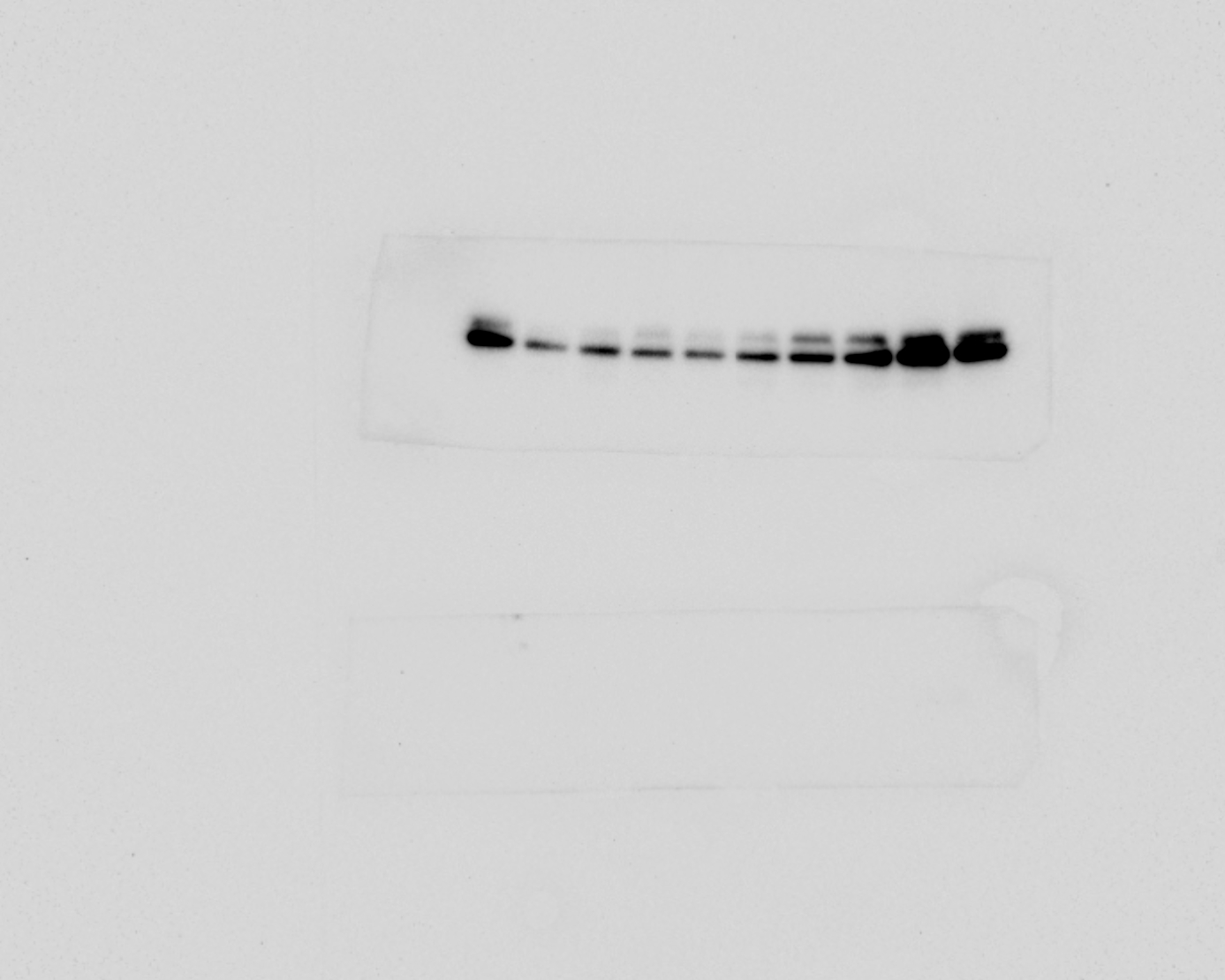

Supplement: Figure 6—source data 3. [file elife-97654-fig6-data3.zip › Figure 6-sourse data3/2022-09-15 Fig6C_S6.jpg]

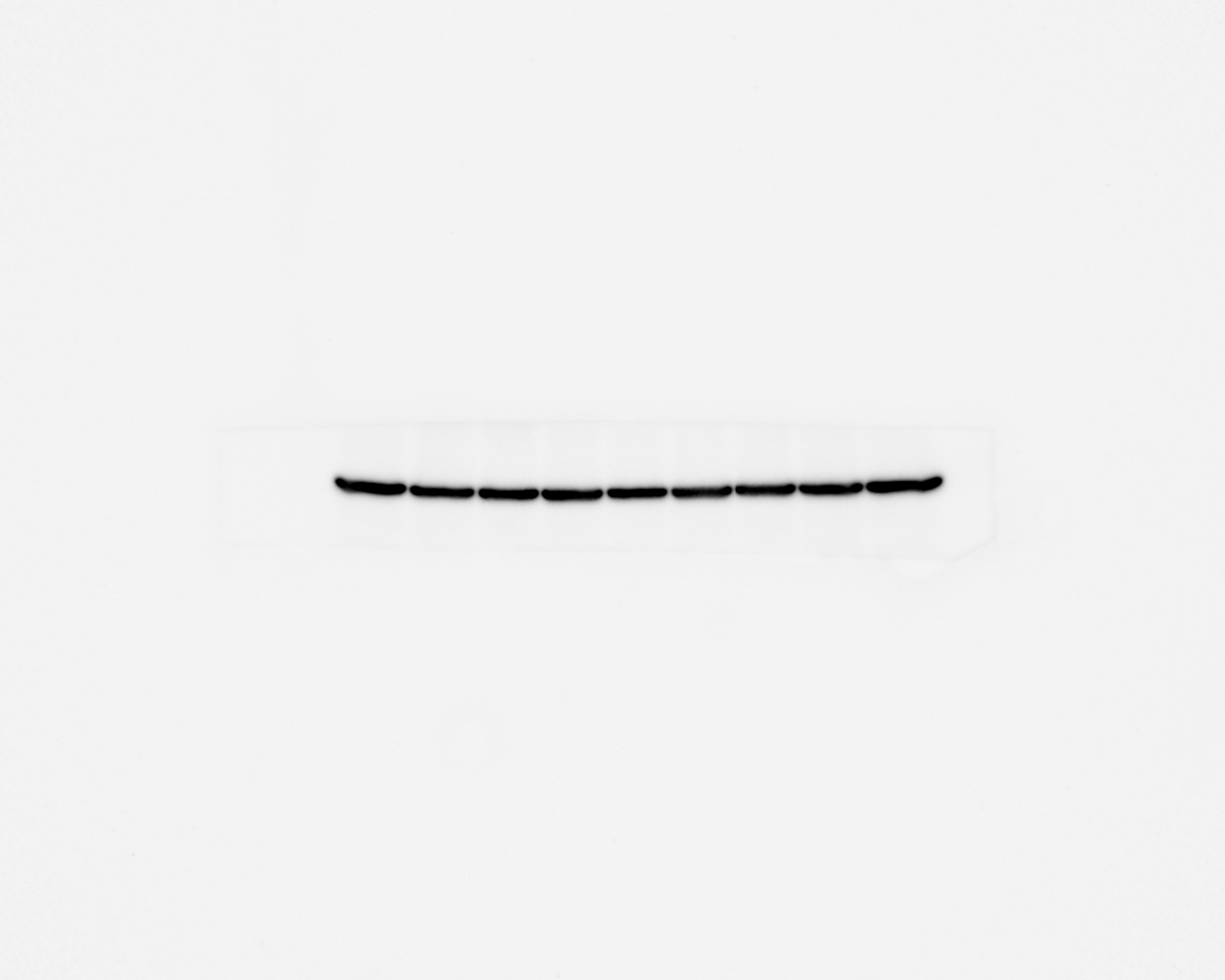

Supplement: Figure 6—source data 3. [file elife-97654-fig6-data3.zip › Figure 6-sourse data3/2023-01-24 Fig6C_bactin for p-Akt and Akt.jpg]

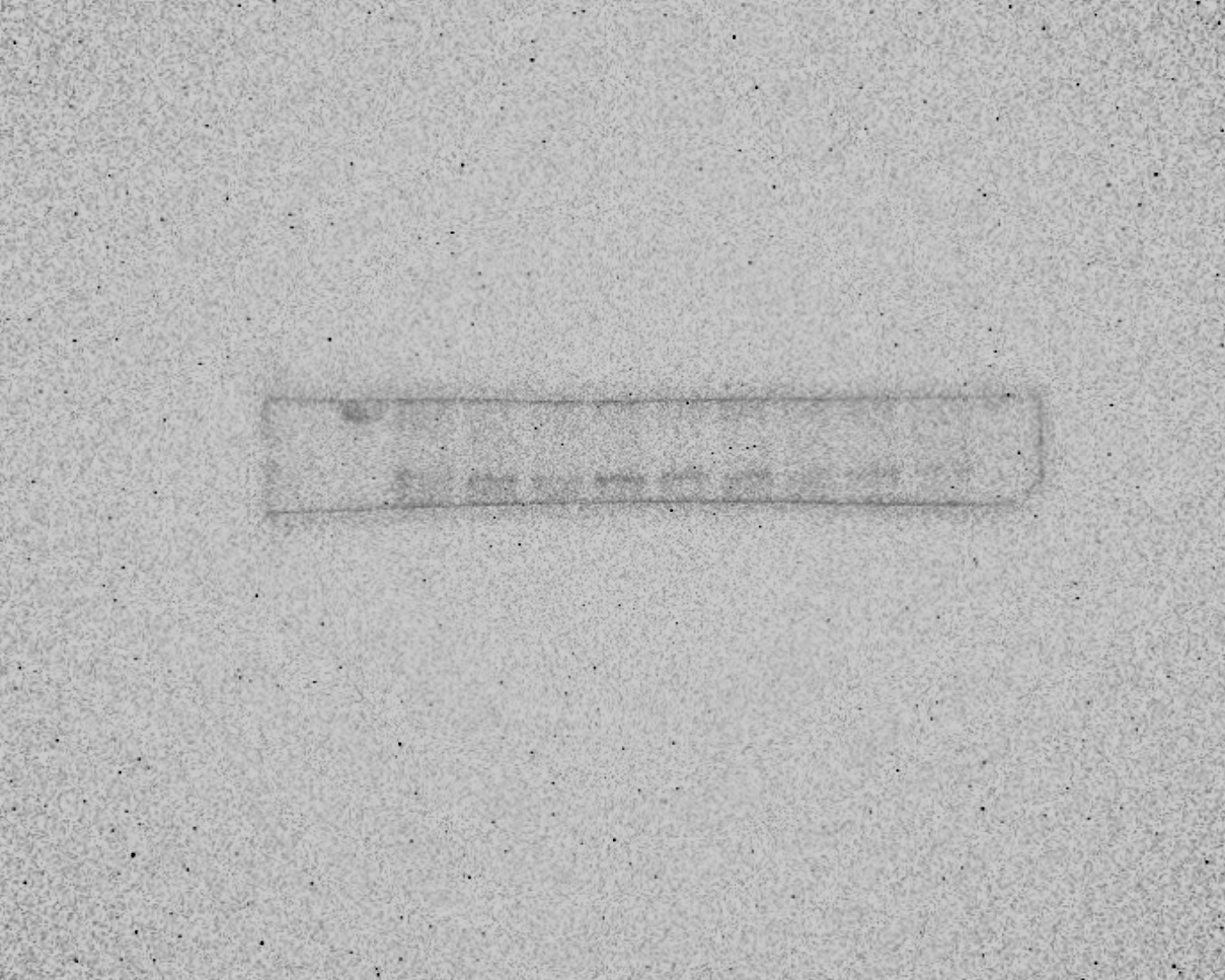

Supplement: Figure 6—source data 3. [file elife-97654-fig6-data3.zip › Figure 6-sourse data3/2023-01-24 Fig6C_p-Akt.jpg]

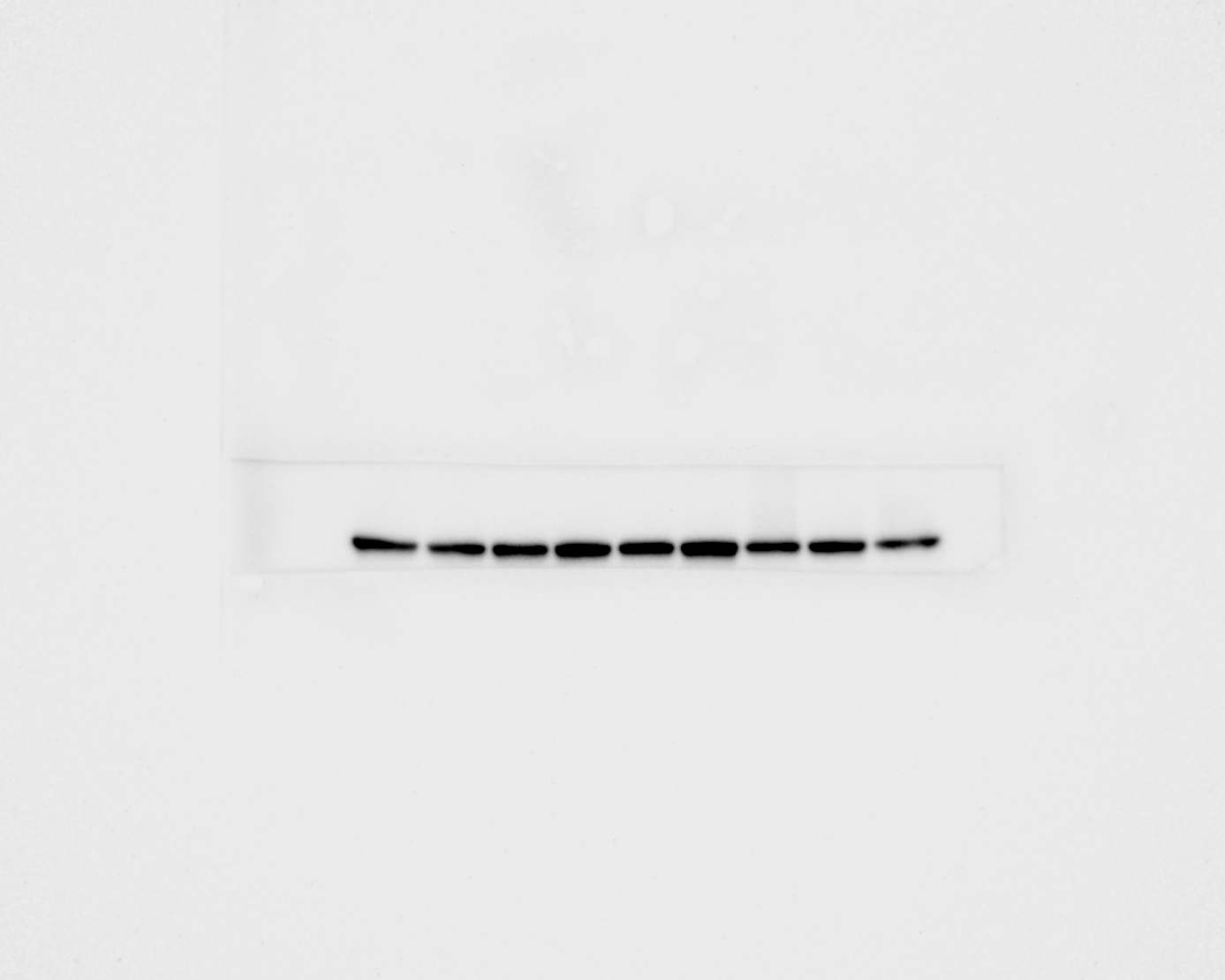

Supplement: Figure 6—source data 3. [file elife-97654-fig6-data3.zip › Figure 6-sourse data3/2023-01-25 Fig6C_Akt.jpg]
